# Evoked resonant neural activity long-term dynamics can be reproduced by a computational model with vesicle depletion

**DOI:** 10.1016/j.nbd.2024.106565

**Published:** 2024-09

**Authors:** James J. Sermon, Christoph Wiest, Huiling Tan, Timothy Denison, Benoit Duchet

**Affiliations:** a Institute of Biomedical Engineering, Department of Engineering Science, University of Oxford, Oxford, UK; b MRC Brain Networks Dynamics Unit, Nuffield Department of Clinical Neurosciences, University of Oxford, Oxford, UK

**Keywords:** Evoked resonant neural activity, Deep brain stimulation, Subthalamic nucleus, parkinson's disease, Synaptic vesicle depletion, Computational modelling

## Abstract

Subthalamic deep brain stimulation (DBS) robustly generates high-frequency oscillations known as evoked resonant neural activity (ERNA). Recently the importance of ERNA has been demonstrated through its ability to predict the optimal DBS contact in the subthalamic nucleus in patients with Parkinson's disease. However, the underlying mechanisms of ERNA are not well understood, and previous modelling efforts have not managed to reproduce the wealth of published data describing the dynamics of ERNA. Here, we aim to present a minimal model capable of reproducing the characteristics of the slow ERNA dynamics published to date. We make biophysically-motivated modifications to the Kuramoto model and fit its parameters to the slow dynamics of ERNA obtained from data. Our results demonstrate that it is possible to reproduce the slow dynamics of ERNA (over hundreds of seconds) with a single neuronal population, and, crucially, with vesicle depletion as one of the key mechanisms behind the ERNA frequency decay in our model. We further validate the proposed model against experimental data from Parkinson's disease patients, where it captures the variations in ERNA frequency and amplitude in response to variable stimulation frequency, amplitude, and to stimulation pulse bursting. We provide a series of predictions from the model that could be the subject of future studies for further validation.

## Highlights

• Long-term ERNA dynamics can be modelled using a single neural structure.
• The proposed model captures the long-term characteristics of ERNA with variable neurostimulation paradigms.
• Synaptic vesicle depletion is one the key features contributing to long-term ERNA dynamics in the model.

## 1 Introduction

Deep brain stimulation (DBS) of the subthalamic nucleus (STN) has been a widely used treatment for Parkinson's disease (PD) for over two decades and has proven effective for the alleviation of Parkinsonian motor symptoms. During STN-DBS, evoked resonant neural activity (ERNA) is commonly observed in simultaneous recordings of the STN. However, the underlying mechanisms of this phenomenon are not well understood.

ERNA is a series of high-frequency oscillations (HFOs, >200Hz) of high-amplitude in response to 70-180 Hz stimulation, despite there being very little to no high frequency activity at the stimulation site pre-stimulation (Wiest et al., 2020) (also see 4 in Fig. 3A). ERNA has been observed in different neural structures, but so far only in the pallidal (Schmidt et al., 2020; Johnson et al., 2023) and subthalamic regions (Wiest et al., 2020; Sinclair et al., 2018; Sinclair et al., 2019; Wiest et al., 2023). Furthermore, ERNA has also been characterised as a robust response across different devices, patient groups and stimulation conditions (Wiest et al., 2020; Sinclair et al., 2018; Sinclair et al., 2019; Wiest et al., 2023). However, the reproducibility and unphysiological appearance of ERNA have led to questions of whether it is of biophysical origin. The biophysical origin of ERNA was later demonstrated in (Sinclair et al., 2019), but many questions regarding the mechanisms of ERNA remain.

Since Sinclair et al. observed the presence of ERNA in the Parkinsonian STN (Sinclair et al., 2018), several hypotheses and models looking to explain the presence of ERNA have been proposed. These hypotheses range from inhibition and/or excitation of neural ensembles to recruitment of inter-component projections between the STN and globus pallidus externus (GPe) (Wiest et al., 2023; Chiken and Nambu, 2016; Milosevic et al., 2018; Steiner et al., 2019). However, thus far none of these propositions have managed to explain all ERNA characteristics, in particular the long term variation of ERNA with continuous DBS. Recently, the significance of comprehending the fundamental mechanisms of ERNA has become more apparent with evidence that ERNA may provide the strongest predictor of clinical performance for STN-DBS contact selection (San San et al., 2022).

In this study, we propose a biophysically-motivated computational model that can reproduce the key long-term ERNA characteristics currently described in the literature under a variety of STN-stimulation paradigms, with a view to hypothesise the fundamental mechanisms responsible for ERNA. We assembled a single population of Kuramoto oscillators intended to represent the STN. Through the addition of synaptic vesicle depletion and a second-order coupling function to the standard Kuramoto model, we were able to fit the parameters of the modified Kuramoto model to key features of ERNA. We simulate further stimulation paradigms to validate and gain insights into the key properties of the computational model. In addition, we suggest predictions that can be used to further validate the computational model.

## 2 Methods

In this section, we first provide an overview of the computational model, starting with an introduction to the Kuramoto model as well as the details of the modifications to the model, in particular the effect of vesicle depletion and replenishment on coupling strength. We then present the fitting process used to find model parameters that reproduce key characteristics of the slow dynamics of ERNA. Lastly, we describe methodological details for all simulations performed in this study based on different experimental paradigms.

### 2.1 The Kuramoto model

Our approach is to attempt to model the ERNA using as simple a model as possible. We therefore consider the Kuramoto model, which has been frequently used to represent oscillatory neural activity as well as the effect of DBS (Tass and Majtanik, 2006; Asllani et al., 2018; Weerasinghe et al., 2019; Bick et al., 2020; Weerasinghe et al., 2021; Duchet et al., 2023a), and is one of the simplest models that can describe populations of interconnected neurons. The basic Kuramoto model describes a series of interconnected phase oscillators (Kuramoto, 1983), in our case representing neurons (Weerasinghe et al., 2019; Brown et al., 2004). The evolution of the $i^{\mathrm{th}}$ oscillator's phase $\theta_{i}$ is given by the stochastic differential equation

(1) $$d\theta_{i}=\left[\omega_{i}+k_{t}\sum_{j=1}^{N}f\left(\theta_{j}-\theta_{i}\right)+I_{t}g\left(\theta_{i}\right)\right]\mathrm{dt}+\zeta {\mathrm{dW}}_{i}\;\text{for}\;i=1,\ldots ,N,$$

where $\omega_{i}$ represents the natural frequency of the $i^{\mathrm{th}}$ oscillator. The (homogeneous) coupling strength between oscillators at time t is $k_{t}$, and f represents the coupling function, which is a 2π-periodic function of phase differences. The phase response of individual oscillators is described by the 2π-periodic phase response curve (PRC) which is denoted g, and $I_{t}$ is the intensity of stimulation at time t modelled as

(2) $$I_{t}=I\cdot I_{\text{fact}}\cdot u\left(t\right),$$

where I is the fitted stimulation amplitude and $I_{\text{fact}}$ is a factor included to vary stimulation amplitude. The stimulation pulse train u at time point $t_{i}$ represents a simplified DBS pulse train obtained as

$$u\left(t_{i}\right)=\left\{\begin{array}{cc} 1 & \text{if}\left(t_{i}\;\mathrm{mod}\;\frac{1}{f_{\text{stim}}}\right)<\Delta t,\\ 0 & \text{otherwise}, \end{array}\right)$$

where $f_{\text{stim}}$ is the stimulation frequency and Δt the simulation time step. We are assuming that all oscillators perceive the same level of stimulation. Intrinsic noise is added to each oscillator through independent Wiener processes $W_{i}$. The noise standard deviation is set by ζ. The number of Kuramoto oscillators in the model is given by N, which is chosen to be 50 for this study unless stated otherwise. Numerical studies of the Kuramoto model in neuroscience are commonly on the scale of 20 to 100 oscillators (Tass and Majtanik, 2006; Asllani et al., 2018; Duchet et al., 2023a; Duchet et al., 2023b). We thus chose 50 oscillators as the lower end of a large population size. This number of oscillators allowed us to perform the computationally intensive optimisations required for this study, while providing comparable results to a larger network. To measure synchrony in the network, we use the order parameter given by

(3) $$Z=\frac{1}{N}\sum_{j=1}^{N}e^{i\theta_{j}}.$$

It is necessary to introduce a series of modifications to the Kuramoto model in order to incorporate dynamics that can adequately capture the ERNA.

### 2.2 Modelling the effect of vesicle depletion and replenishment on coupling strength

Vesicle depletion and progression towards synaptic failure has been suggested as an underlying mechanism of the ERNA frequency decay (Wiest et al., 2023). However, no clear mechanism describing how vesicle depletion may influence the dynamics of ERNA has been proposed. We will develop the vesicle depletion hypothesis in this study and sketch how it could explain the underlying dynamics of ERNA.

**Fig. 1:**
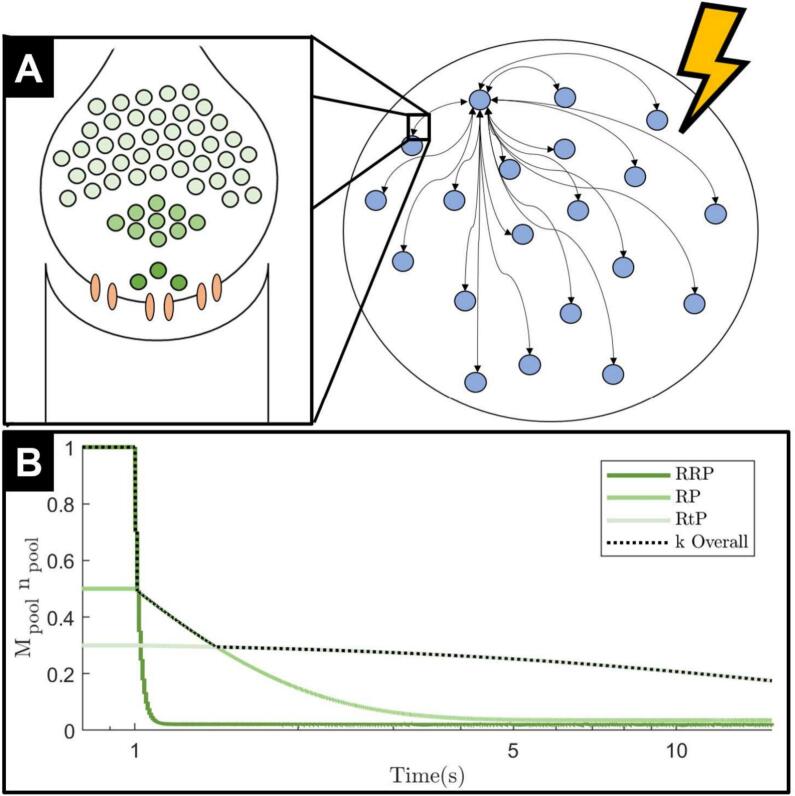
Modified Kuramoto model. (A) Right: One population of all-to-all connected Kuramoto oscillators (blue circles), which all receive stimulation of the same magnitude. Only showing bi-directional connections to one example oscillator. Left insert: We zoom in on the synaptic connections between each oscillator which are affected by vesicle depletion. The closest vesicle pool to the synaptic terminal is the RRP (darkest green), followed by the RP (lighter green). The pool furthest away from the synaptic terminal is the RtP (lightest green). Vesicle pool colours are consistent with colours used in panel B. (B) Coupling strength decay curve, with stimulation starting at 1 s. The three green colours represent the contribution of each of the three vesicle pools. The black dotted line is the output of the maximum function in Eq. 5, which dictates the overall coupling strength. (For interpretation of the references to colour in this figure legend, the reader is referred to the web version of this article.)

Rizzoli and colleagues proposed the three-vesicle pool model of synaptic transmission (see Fig. 1A) (Rizzoli and Betz, 2005). Firstly, the readily-releasable pool (RRP) is a small but easily-mobilised group of vesicles. Due to their proximity to the synaptic terminal and small number, RRP vesicles are often depleted within a second of stimulation onset and recycle within seconds. Secondly, the recycling pool (RP) is a slightly larger pool of vesicles which are less easily mobilised, but still deplete and recycle within seconds. Finally, the reserve or resting pool (RtP) represents the majority of vesicles at the synaptic terminal. The RtP is the least easily mobilised vesicle pool, and its vesicles are depleted over minutes (depletion only occurs under strong stimulation) and recycle over a similarly long time period. The vesicle pools are recruited in this sequential order. The RRP is the primary vesicle pool responsible for synaptic transmission under physiological firing conditions. It is able to recycle vesicles between action potentials under physiological conditions and was first observed as quanta docked to the synaptic terminal (Rosenmund and Stevens, 1996).

In the context of ERNA, however, the STN is experiencing unphysiological stimulation above the threshold required to observe vesicle depletion (Fernández-Alfonso and Ryan, 2004). Under these conditions, simulations show the RRP depleted within approximately 10 pulses of stimulation at 100 Hz (Kaeser and Regehr, 2017). Hence, after prolonged exposure to unphysiological stimulation the majority of the vesicles responsible for synaptic transmission will belong to the RtP (Denker Annette and Rizzoli, 2010; Abdul and Alabi and Richard W. Tsien., 2012). During the early stages of stimulation and vesicle depletion, the number of vesicles remaining at the terminal appears to follow an exponential decay (Abdul and Alabi and Richard W. Tsien., 2012). Vesicle depletion of STN axonal efferents has been observed in response to STN-DBS in both in vivo and in vitro recordings (Rosenbaum et al., 2014).

Computational models characterising the effect of vesicle depletion on synaptic connectivity and the response of a vesicle pool to stimulation have previously been developed (Hennig and Matthias, 2013; Peterson et al., 2014). We employ the first order kinetic model used in (Hennig and Matthias, 2013) to track vesicle pool occupancy. Given the time scale differences over which each pool depletes and replenishes, we model the dynamics of each pool independently. This simplified approach is sufficient to capture the three different time constants of each vesicle pool (see Fig. 1B) as seen in data (Rizzoli and Betz, 2005). Following (Hennig and Matthias, 2013), we model the occupancy of each vesicle pool $n_{\text{pool}}\in \left[01\right]$ (with $\text{pool}\in \left\{\mathrm{RRP},\mathrm{RP},\mathrm{RtP}\right\}$) as

(4) $$\frac{dn_{\text{pool}}}{\mathrm{dt}}=\frac{1-n_{\text{pool}}\left(t\right)}{\tau_{\text{pool}}}-\sum_{j}\delta \left(t-t_{j}\right)\cdot p_{\text{pool}}\cdot n_{\text{pool}}\left(t\right),$$

where $\tau_{\text{pool}}$ is the time constant of replenishment and $p_{\text{pool}}$ is the probability of vesicle release in response to a stimulation pulse for the fitted stimulation amplitude for each vesicle pool. The values used for these parameters are estimated from the time taken to deplete and replenish each of the vesicle pools, respectively, according to data (Rizzoli and Betz, 2005; Kaeser and Regehr, 2017) – see Section A in Supplementary Material. In modelling the evolution of pool occupancy, we focus on the effect of stimulation and ignore spikes not triggered by stimulation. Thus, $\delta \left(t-t_{j}\right)$ is a Dirac function where the $t_{j}$’s correspond to the time points of stimulation.

We model the effect of depletion and replenishment of the three vesicle pools (given by Eq. (4)) on coupling strength based on three assumptions. First, activity at a synaptic terminal directly corresponds to the number of vesicles released (Chamberland and Tóth, 2016), and if fewer vesicles are released into the synapse, the effect of pre-synaptic activity on downstream neurons will be weaker. Thus, we model the effect of vesicle dynamics on coupling strength as proportional to the number of vesicles available.

Second, we translate the occupancy of each vesicle pool into its effect on coupling strength using a factor $M_{\text{pool}}$, which we estimate for each vesicle pool using data as the product of both the pool's relative size (Rizzoli and Betz, 2005) and the inverse of its approximate distance from the synaptic terminal (Rizzoli and Betz, 2005; Lou et al., 2012; Branco and Staras, 2009; Lei et al., 2009; Staras et al., 2010; Südhof, 2012; Dittman and Ryan, 2019) (less readily-releasable vesicles further from the synaptic terminal have a lower release probability for a given stimulation (Lou et al., 2012)). The values used for the pool-coupling factors, $M_{\text{pool}}$’s, are given and justified in Section A in Supplementary Material.

Third, at any point in time, we only consider the effect on coupling strength of the dominant pool (largest $M_{\text{pool}}\cdot n_{\text{pool}}\left(t\right)$ at time t). This reflects the sequential nature of vesicle pool depletion and how one pool tends to dominate synaptic transmission at any point in time depending on depletion levels. It also allows the vesicle depletion curve to take the form of an exponential decay with three time constants (Fig. 1B) as seen in (Rizzoli and Betz, 2005). Moreover, while on-stimulation coupling strength is impacted (in succession) by the depletion of each vesicle pool, the recovery of coupling strength following stimulation termination is chiefly determined by the replenishment of the RRP. Other vesicle pools do not appear to play a significant role as synaptic activity recovers at a similar rate regardless of the stimulation parameters used during the stimulation window (Fernández-Alfonso and Ryan, 2004) (i.e. regardless of how depleted the other pools are), which is consistent with this third assumption. Since the RRP is replenished much faster than other pools (within seconds following stimulation termination (Rizzoli and Betz, 2005)), the RRP quickly resumes being the primary vesicle pool for synaptic transmission.

Based on these three assumptions, the coupling strength at time t is obtained as

(5) $$k_{t}=k_{\mu }\cdot \max\left(M_{\mathrm{RRP}}\cdot n_{\mathrm{RRP}}\left(t\right),M_{\mathrm{RP}}\cdot n_{\mathrm{RP}}\left(t\right),M_{\mathrm{RtP}}\cdot n_{\mathrm{RtP}}\left(t\right)\right),$$

where $k_{\mu }$ is the steady state coupling strength of the network off stimulation.

### 2.3 Coupling function and PRC

In addition to the modulation of coupling strength over time when the network is exposed to stimulation, we incorporate a second-order Fourier series coupling function f into the model and fit its coefficients $\left(f_{i}\right)$, $i\in \left\{0,..,4\right\}$ to data. The coupling function is thus given by

(6) $$f\left(x\right)=f_{0}+f_{1}\cos\left(x\right)+f_{2}\sin\left(x\right)+f_{3}\cos\left(2x\right)+f_{4}\sin\left(2x\right).$$

Compared to a sinusoidal or Hodgkin-Huxley-derived coupling function (explored in Supplementary Material Section D.1), this more general coupling function allows the model to capture more complex interactions between neurons.

For the majority of this work, we consider a sinusoidal PRC, i.e. $g\left(x\right)=\sin\left(x\right)$. The inclusion of a second-order PRC was also considered, as well as sinusoidal and a Hodgkin-Huxley PRC (see Supplementary Material Section D.2). The form of the PRC was not as crucial to the intricate dynamics of ERNA as the coupling function was observed to be.

### 2.4 Distributions of oscillators' natural frequency

Since the STN is a nonuniform neuronal structure, oscillator's natural frequencies are sampled from a normal distribution, $\omega_{i}∼N\left(\Omega ,\sigma^{2}\right)$. We do not constrain the mean angular frequency Ω and standard deviation σ to a pre-determined narrow frequency band in the fitting process to provide added versatility to the model dynamics. Some in vivo and in vitro recordings of STN neurons showed firing rates of around 20 Hz off stimulation (Hallworth et al., 2003; Wilson et al., 2004). However, these recordings are from networks of neurons and may not represent isolated neurons' intrinsic firing rates. This may be supported by the observation of some high-frequency activity off stimulation in the STN (see 4 in Fig. 3A). We present simulations with natural frequencies constrained to 20 Hz in Supplementary Materials Section F.

### 2.5 Fitting process

In order to find a set of parameters for the modified Kuramoto model that yield dynamics similar to ERNA, we fit the model to features of the slow dynamics of ERNA. The fitting methodology used in this study is similar to other computational studies (Duchet et al., 2020; Duchet et al., 2021; Sermon et al., 2023) and uses the patternsearch MATLAB function to minimise a cost function representing the distance between the data features and the same features calculated in the model. The Kuramoto model was simulated using a Euler-Maruyama scheme with a timestep $\Delta t=10^{-4}$.

**Fig. 2:**
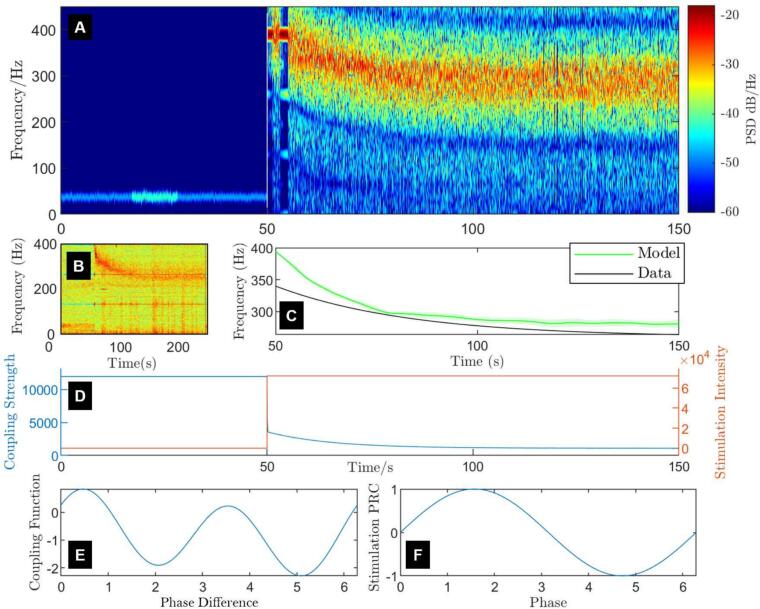
Fitted slow dynamics of the modified Kuramoto model on and off stimulation and model's key properties. (A) Spectrogram showing the fitted model's ERNA response to 130 Hz stimulation (fitted stimulation amplitude) over time. The transition from off to on stimulation happens at 50 s. The colour scale represents the power spectral density (PSD). (B) Example of the slow ERNA dynamics in the frequency domain for 130 Hz and 3 mA stimulation adapted from (Wiest et al., 2020). (C) Comparison of the peak ERNA frequency decay estimated from panel B and fed into the fitting process (black), and the model predicted ERNA frequency peak (green) averaged over 15 realisations of noise, natural frequencies and initial conditions for the 100 s period of stimulation following 50 s of the model settling off-stimulation. We also display the standard error of the mean for the averaged model output in the lighter green band. (D) Evolution of coupling strength in the model (blue) and stimulation intensity (orange) over the full stimulation window. Stimulation is a pulse train at 130 Hz, but intensity is depicted as constant following stimulation onset in this figure for demonstrative purposes. (E) The fitted second-order Fourier series coupling function, and (F) the phase-response curve (PRC, here a sine wave) used in the model. (For interpretation of the references to colour in this figure legend, the reader is referred to the web version of this article.)

The cost function used in the optimisation process is calculated from the on-stimulation ERNA frequency decay (slow dynamics, Fig. 2B). An approximation of the peak frequency of the ERNA decay (the change in the maximum frequency in each sampling window following stimulation onset) in the STN for a trial of 130 Hz and 3 mA STN DBS (patient on medication) was obtained using Fig. 2B (taken from (Wiest et al., 2020)) as

(7) $$f_{\text{ERNA}}^{\mathrm{dat}}\left(t\right)=260+80e^{-0.07t}\mathrm{Hz},$$

where t is the time elapsed since DBS was turned on. The estimate $f_{\text{ERNA}}^{\mathrm{dat}}$ is represented by the black line in Fig. 2C. The Kuramoto model was then simulated for a period of 100 s on stimulation following a 20 s settling period off stimulation. The model's frequency decay $f_{\text{ERNA}}^{\mathrm{mod}}$ was calculated as the maximum power spectral density (PSD) frequency between 240 and 400 Hz for a series of one 100 ms windows within the 100 s stimulation period. The cost of this feature was obtained as

(8) $${\text{Cost}}_{\mathrm{On}}=\sum_{i=1}^{n_{t}}\left|f_{\text{ERNA}}^{\mathrm{mod}}\left(t_{i}\right)-f_{\text{ERNA}}^{\mathrm{dat}}\left(t_{i}\right)\right|,$$

where the $t_{i}$’s correspond to sampling every 100 ms and $n_{t}$ is the number of time samples.

Another spectrogram feature was used to limit high frequency activity before stimulation. This was done by calculating the maximum over time off stimulation of the complex modulus of the order parameter, ${\left|Z\right|}_{\max}$, a metric for the synchronisation of the oscillators in the network. The off-stimulation cost was defined as

(9) $${\text{Cost}}_{\mathrm{Off}}=\left\{\begin{array}{ll} 0 & \text{if }{\left|Z\right|}_{\max}<0.2,\\ \left({\left|Z\right|}_{\max}/0.2\right)-1 & \text{otherwise}, \end{array}\right)$$

where the value 0.2 acts as a threshold under which the off-stimulation cost is zero, and above which the off-stimulation cost grows linearly with ${\left|Z\right|}_{\max}$. The threshold of 0.2 provided enough of a contrast compared to on-stimulation activity, which tended to be much closer to 1.

The on-stimulation feature was chosen as the most important to replicate, and considering the scale of both features, the total cost corresponding to the slow dynamics of ERNA was evaluated as

(10) $$\text{Cost}=0.9{\text{Cost}}_{\mathrm{On}}+0.1{\text{Cost}}_{\mathrm{Off}}.$$

### 2.6 Simulation of various stimulation paradigms

To ascertain the computational model's suitability for investigating ERNA, we validate its performance by subjecting it to various stimulation paradigms that replicate conditions explored across a range of experimental studies.

### 2.7 Long-term spectrogram

**Fig. 3:**
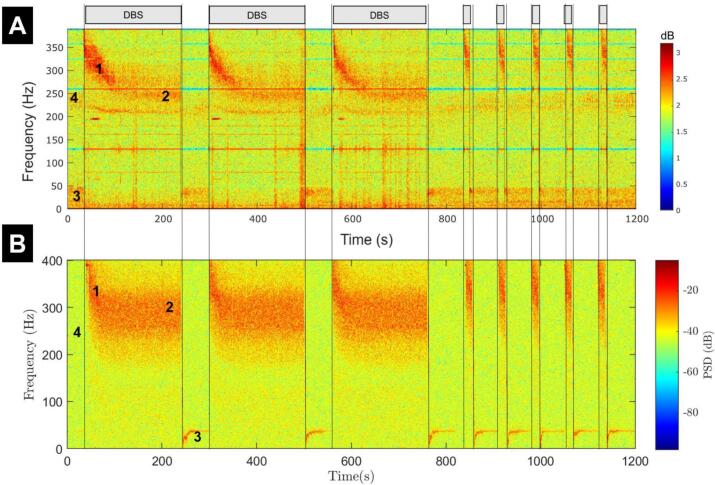
The modified Kuramoto model replicates ERNA characteristics observed in data during multiple on-DBS windows with pauses. (A) Data spectrogram, adapted from (Wiest et al., 2020). On-DBS periods (with 130 Hz, 3 mA STN DBS) are indicated by grey rectangles. (B) Spectrogram from the modified Kuramoto model with added white noise. Both panels use similar time points of DBS initiation and termination. Annotations are described in the main text. Since the model output is on a very different scale from the data, the scale of the model output PSD differs from that of the data.

We replicated the long-term spectrogram reported in (Wiest et al., 2020), where DBS is turned on and off in a series of periods of varying lengths for a total of 1200 s of continuous observations (Fig. 3A). The on- and off-stimulation period durations were chosen to align most closely with the data (Fig. 3A). All stimulation periods are at 130 Hz and at the same stimulation amplitude as the model was fitted to (3 mA). The simulation is initialised with all vesicle pools operating at 100% occupancy. White noise is added to the order parameter (extrinsic noise) using the ‘awgn’ function of MATLAB with a signal-to-noise ratio of 20 (chosen by visual inspection to resemble the background activity of the data).

### 2.8 Variable stimulation amplitude

In order to replicate ERNA dynamics in the model under various stimulation amplitudes, adjustments to the model have to be made. We provide simple, biophysically-motivated changes to the model and fit the changes by manual parameter tuning to the ERNA frequency in the data (Wiest et al., 2023) at the end of the stimulation window. To validate these changes, we then compare the amplitude of ERNA in the model to that of the data.

Unlike stimulation frequency, the scale of stimulation amplitude in the model cannot be directly compared to the stimulation amplitude used in the data. This makes interpretation of the stimulation amplitude range more difficult. We assume that increasing stimulation amplitude will increase the rate of vesicle depletion by depleting more vesicles in response to each stimulation pulse, which corresponds to an increase in $p_{\text{pool}}$. Increasing stimulation amplitude should therefore result in similar effects to increasing stimulation frequency in data (Schor and Nelson, 2019). We model the effect of changing stimulation amplitude by replacing $p_{\text{pool}}$ in Eq. (4) by

(11) $$p_{\text{pool},I_{\text{fact}}}=p_{\text{pool}}\cdot I_{\text{fact}},$$

where $I_{\text{fact}}$ is the stimulation amplitude modulation factor (see Eq. (2)). This linear model is only appropriate for $I_{\text{fact}}$ close to one. In Fig. 5, $I_{\text{fact}}$ is between 0.8 and 1.1 (with $I_{t}$ given by Eq. (2)).

### 2.9 Evaluation of ERNA frequency and amplitude over time

We visualise the computational model’s output in a manner consistent with the approaches used in the corresponding experimental paradigms (Wiest et al., 2023). We consider the real part of the order parameter Z to approximate the neuronal signals from data, in line with (Weerasinghe et al., 2019). Spectrograms are calculated both on and off stimulation using the ‘pwelch’ MATLAB function applied to the real part of Z over one second windows with a 25% overlap for Fig. 3 (in line with (Wiest et al., 2020)) and 100 ms windows with a 50% overlap everywhere else as these are shorter simulations. While analysing the model over these timescales could result in missing fast ERNA dynamics, the parameters of this analysis were chosen to provide comparable results to the data. Furthermore, we have checked that processing the model's output with a window duration matching the stimulation period and without overlap produces results with little difference to the key features recognised in this analysis. There are no notch filters in place at stimulation frequency or its harmonics as the artefacts of stimulation in the real part of Z of our model are relatively small or nonexistent. The ERNA frequency response over time is calculated by finding the frequency of maximum PSD on stimulation in one second windows. ERNA amplitude is calculated by taking the complex modulus of Z. This is by definition (Eq. (3)) below one, thus the range of ERNA amplitudes cannot be directly compared between the model and the data. However, trends and timing of the amplitude dynamics can be used for comparison. In the data, each second a pulse is skipped to allow for the frequency and amplitude of the evoked response to be recorded. While the model does not suffer from the stimulation artefact obscuring the evoked response, we take a similar approach and average both ERNA frequency and amplitude over moving windows of one second to match the windowing used in the data.

## 3 Results

We modified the Kuramoto model to include a second-order coupling function as well as the effect of vesicle depletion from high-frequency DBS, and fitted the modified model to salient long-term features of ERNA observed in patient data with DBS. By comparing simulations of the fitted model to ERNA frequency responses (frequencies of maximum PSD for each sampling window of the evoked signal) recorded in patients under varying stimulation frequencies, and with minimal additional parametrisation under varying stimulation amplitudes, we characterise the model and test its predictive power. We provide validation for the model by comparing the model's ERNA amplitude response (the complex modulus of Z), which is not considered in the parameter tuning, to that of the data. It should be noted that the authors were not blinded to the data used in this study, therefore further validation of the model using unseen data is still warranted.

### 3.1 Results of fitting process and key factors contributing to ERNA in the model

The best-ranked parameter set resulting from the fitting to the peak frequency ERNA decay while limiting off-stimulation high-frequency activity provided a good approximation of the peak frequency ERNA decay estimated from data recorded in a PD patient with 130 Hz, 3 mA STN DBS (Fig. 2C). The corresponding model parameters are given in Table A in Supplementary Material. In the spectrogram, the fitted model shows a broad ERNA decay approximating patient data (compare Fig. 2A and Fig. 2B). Before stimulation onset, high-amplitude and high-frequency activity is absent in the model, with a lower amplitude peak at around 40 Hz as in the data (compare Fig. 2A and characteristic 3 in Fig. 3A). We detail the underlying model dynamics that produce the model's amplitude and frequency changes in Supplementary Material section B. Following stimulation onset, we note two short periods of entrainment at the third harmonic of stimulation frequency (390 Hz). This is caused by the strong synchronising effect of stimulation and the oscillating frequency of the network being close to the third harmonic of stimulation frequency.

The first key factor that was found to be necessary for reproducing the slow ERNA dynamics in the model is the reduction of coupling strength through vesicle depletion during stimulation (Fig. 2D). Over the timescale during which the slow ERNA dynamics develop, the RRP as well as the RP are very quickly exhausted, and the RtP becomes the dominant vesicle pool for synaptic transmission (Rizzoli and Betz, 2005; Fernández-Alfonso and Ryan, 2004; Kaeser and Regehr, 2017). Although larger, the RtP is the furthest away from the synaptic terminal and unable to provide vesicles to facilitate synaptic transmission as readily as other pools (Rizzoli and Betz, 2005; Denker Annette and Rizzoli, 2010; Abdul and Alabi and Richard W. Tsien., 2012). We model this effect as a reduction in coupling between neurons. Hence, Fig. 2D exhibits a fast initial drop in coupling strength as the RRP and RP are quickly depleted, and the RtP becomes dominant.

The second key factor that was found to be necessary for reproducing the slow ERNA dynamics in the model is the second-order Fourier series coupling function. The fitted coupling function (Eq. (1)) has a strong second harmonic as well as a negative shift (Fig. 2E). We show in Supplementary Material Section D.1 that neither a coupling function derived from the Hodgkin-Huxley model, nor a sine coupling function are suitable to reproduce the slow ERNA dynamics. We also demonstrate in Supplementary Material Section D.1 that the zeroth and second harmonic of the coupling function have a larger impact on the ERNA frequency decay than the first harmonic. The importance of the second harmonic is related to the clustering behaviour exhibited by the model (see Section B in Supplementary Material). In the Kuramoto model, stimulation can advance or delay a neuron's spiking by advancing or delaying the phase of individual oscillators. This effect is captured by the PRC (g in Eq. (1)), which characterises the change in an individual oscillator's phase as a function of the phase at the time of stimulation. For this parameter set, a sine PRC (Fig. 2F) was sufficient to provide an accurate fit to the features, which further limited the number of parameters that needed to be optimised.

The third key factor that was found to be necessary for reproducing the slow ERNA dynamics in the model is that oscillators have an intrinsic ability to fire at much higher rates than the off-stimulation network firing rate. We have repeated the fitting process with natural frequencies constrained to lower frequencies (20 Hz) and were unable to reproduce long-term ERNA dynamics (more details in Supplementary Material Section F). We also look to support this observation through comparisons of neuronal firing rate in largely disconnected STN slice experiments in Supplementary Material Section E.2.

We do not constrain model parameters using any short-term ERNA feature (see Methods Section *Fitting Process*), which is why the model does not capture fast ERNA dynamics well. We discuss this in greater detail in the Discussion Section 4.4.1. and Supplementary Material Section C.1.

### 3.2 Long-term spectrogram

While Fig. 2A captures the slow dynamics of ERNA over more than a minute on-stimulation, Wiest et al. provide a series of on-DBS windows and pauses that demonstrate how reproducible ERNA is (Wiest et al., 2020). Additionally, Fig. 2A does not demonstrate the ability of the model to return to baseline following stimulation, a key observation of ERNA dynamics. We address this by simulating the same DBS on/off paradigm used in (Wiest et al., 2020) and depicted in Fig. 3A. The spectrogram (with the addition of white noise) appears similar to that of the data (compare Fig. 3A and Fig. 3B), and consistently returns to baseline after stimulation is turned off.

Wiest et al. outline four key characteristics of the ERNA spectrogram, see annotations in Fig. 3A: (1) Initial high frequency, high amplitude response; (2) Steady state reached at a peak of approximately 250 Hz after around 100 s of stimulation; (3) Activity off-stimulation at the low gamma/high beta frequency range of approximately 40 Hz, and; (4) low amplitude, high frequency activity at around 250 Hz off-stimulation (Wiest et al., 2020). The long-term spectrogram from the model (Fig. 3B) demonstrates most of these characteristics and is capable of explaining all of them through model dynamics. Characteristics 1, 2, and 3 can each be seen in the spectrogram regardless of the length of the DBS window. Characteristic 3 is slightly more finely tuned in the model, but occurs at a comparable frequency to the data (except before the first stimulation window). Characteristic 4 cannot be seen in the model's spectrogram. However, this is approximately the mean natural frequency of the oscillators in the model, which could account for characteristic 4.

### 3.3 Variable stimulation frequency

**Fig. 4:**
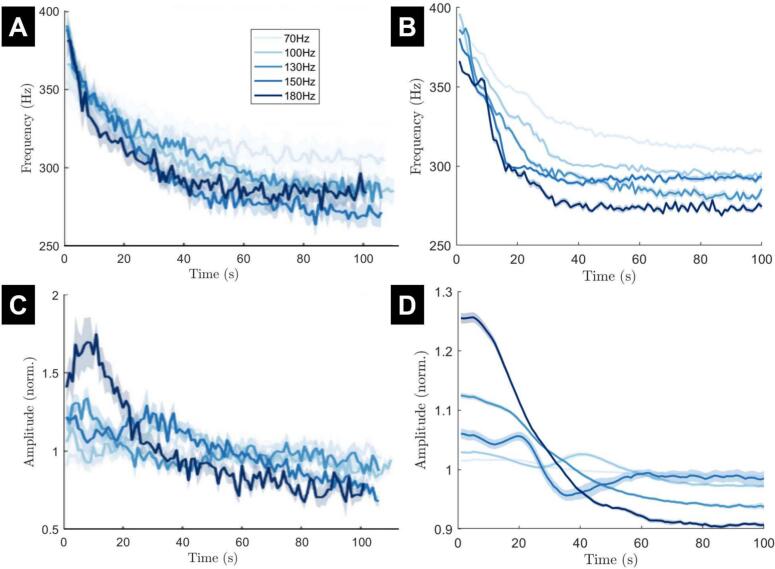
The frequency and amplitude of ERNA over time in response to various stimulation frequencies in both data and the fitted model. (A and C) The data ERNA frequency decay and amplitude variation, respectively. Stimulation amplitude was maintained at the patient's therapeutic intensity. (B and D) The ERNA frequency decay and amplitude variation, respectively, from model simulations. The 130 Hz line of the model simulation has the same parameters as the simulation from Fig. 3. Mean (solid line) and standard error of the mean (faded region around solid mean) calculated over 15 trials with different realisations of noise. The colour for each stimulation frequency is the same in data and model panels. Panels A and C are adapted from (Wiest et al., 2023).

We simulate the fitted model for various stimulation frequencies as was done in (Wiest et al., 2023). The variation in stimulation frequency is captured by the delta term in Eq. 4 and will have the effect of depleting all three vesicle pools further and more quickly with higher stimulation frequency. By simply changing the frequency of the pulse train we can replicate most features of the ERNA frequency decay over time at each stimulation frequency reported in (Wiest et al., 2023) (compare Fig. 4A and B). Initial and steady-state frequencies are similar in the data and the model, and lower stimulation frequencies correspond to higher steady state ERNA frequencies in both the data and the model (with the exception of 180 Hz in the data and 150 Hz in the model). The amplitude lines also demonstrate similarities in both cases (Fig. 4C and D). In particular, the 180 Hz stimulation frequency line is initially higher than the other stimulation frequencies and later drops below other stimulation frequencies.

### 3.4 Variable stimulation amplitude

**Fig. 5:**
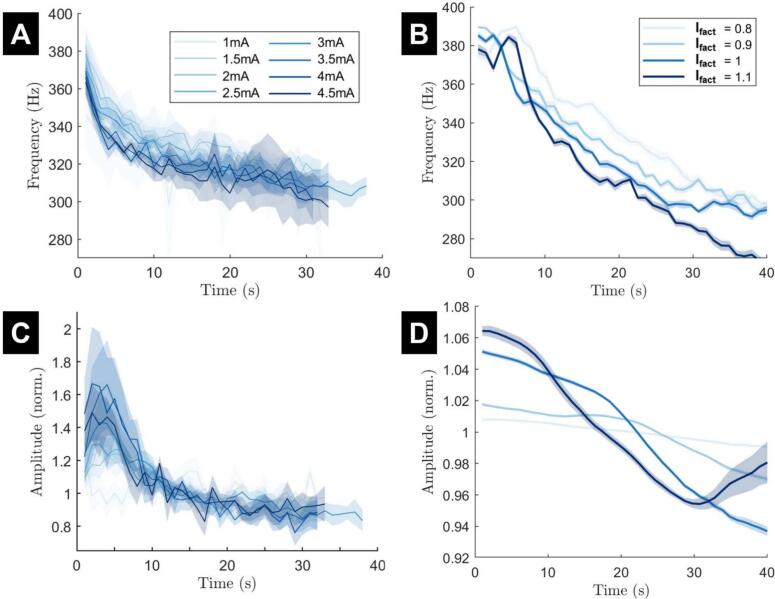
The frequency and amplitude of ERNA over time in response to various stimulation amplitudes in both data and the fitted model. (A and C) The data ERNA frequency decay and amplitude variation, respectively. Stimulation frequency is set at 130 Hz. (B and D) The ERNA frequency decay and amplitude variation, respectively, from model simulations. The $I_{\mathrm{fact}}=1$ line of the model simulation has the same parameters as the simulation from Fig. 3 and approximately corresponds to 3 mA. Mean (solid line) and standard error of the mean (faded region around solid mean) calculated over 15 trials with different realisations of noise. Panels A and C have been adapted from (Wiest et al., 2023).

We also simulate the fitted model for various stimulation amplitudes as was done in (Wiest et al., 2023). Unlike stimulation frequency, the inclusion of variable stimulation amplitude needs to be made through a biophysically-motivated model parameter adjustment as described by Eq. 11 in the Methods section. This adjustment enabled the model to reproduce the ERNA frequency decay at each stimulation amplitude (Fig. 5A and B). Initial and steady-state frequencies are similar in the data and the model (expect for the greatest stimulation amplitude). Higher stimulation amplitudes tend to correspond to lower ERNA frequencies in the data and the model (although the order is less clear in the data due to large error bars). The amplitude lines of the model also demonstrate similarities to the data (Fig. 5C and D). The lowest stimulation amplitude in the model remains relatively flat in the data and the model, while higher stimulation amplitudes show a larger amplitude response. However, differences can also be seen. For instance, the broad peak around four seconds in the data cannot be seen in the model results.

### 3.5 Post stimulation bursting

**Fig. 6:**
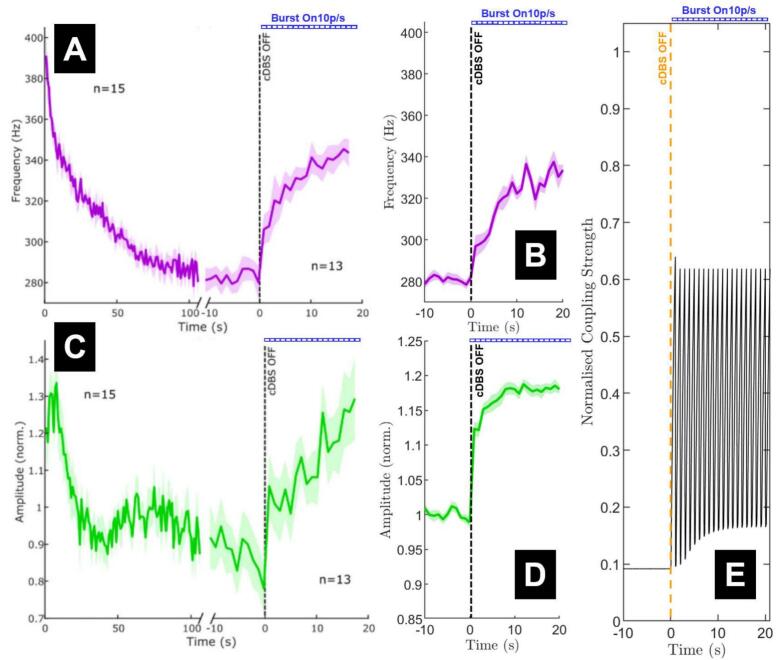
The frequency and amplitude of ERNA in response to bursting stimulation post continuous deep brain stimulation (cDBS) termination in both data and the fitted model. (A and C) The data frequency decay and amplitude variation, respectively. (B and D) The frequency decay and amplitude variation, respectively, from model simulations. Mean (solid lines) and standard error of the mean (faded region around solid mean) calculated over 15 trials with different realisations of noise. (E) Coupling strength normalised by $k_{\mathrm{mu}}$ through continuous stimulation to termination at time 0 (orange dashed line) with 10 pulse bursts at 130 Hz every second for the next 20 s. The blue dashed line in every panel (Burst On 10p/s) indicates the presence of bursting stimulation of 10 pulses at 130 Hz every second following the termination of cDBS. Panels A and C are adapted from (Wiest et al., 2023). (For interpretation of the references to colour in this figure legend, the reader is referred to the web version of this article.)

The responses to post stimulation bursting (10 pulses of stimulation at 130 Hz every second following termination of a long period of stimulation) also appears to show similarities between the model and the data (Fig. 6). This condition captures the cumulative effect of successive, brief periods of vesicle depletion and replenishment (different time scale than in Fig. 3). Vesicle depletion and replenishment over the bursting periods in the model can be visualised in Fig. 6E. In the data, the frequency response increases back up towards the early continuous stimulation state within the 20 s of bursting stimulation (Fig. 6A). A similar increase is seen in the model, reaching approximately the same frequency at similar rates of recovery (Fig. 6B). Amplitude response of ERNA also appears to increase and reach a similar level at the end of the 20 s bursting stimulation window in both the model and the data (Fig. 6C-D). However, the data exhibits a consistent increasing trend in ERNA amplitude over the full 20 s of bursting, while the model only predicts an increase up to around 10 s. The dynamics of these evoked responses change at a rate determined by the vesicle replenishment and coupling strength recovery (Fig. 6E). Coupling strength drops to RtP dominance during each series of stimulation bursts and recovers to RRP dominance between pulse bursts.

## 4 Discussion

In this study, we investigated the ability of a single-population phase oscillator model with no network delays to reproduce the key characteristics of ERNA slow dynamics. We showed that when fitted to an approximation of the ERNA frequency decay obtained from patient data, the model can reproduce the main characteristics of ERNA slow dynamics. One of the key model additions to replicate these characteristics is the inclusion of synaptic vesicle depletion as a result of high-frequency stimulation. The model represents a simplification compared to models previously used to study ERNA, while also being able to reproduce characteristics over longer time periods and in response to a wide range of neuromodulatory perturbations. Importantly, the model demonstrates that ERNA slow dynamics can be reproduced with a single neuronal population without dynamic inputs from other structures. This work highlights the fundamental properties required of a phase-oscillator network to reproduce the slow dynamics of ERNA. This information may prove useful in the development of the clinical understanding of ERNA.

### 4.1 Hypothesised mechanisms and models of ERNA

Several mechanisms have been hypothesised to explain ERNA in STN-DBS (see (Wiest et al., 2023) for a summary). One hypothesis that has gained support is the network between the STN and GPe recurrently modulating activity in both structures. This recurrent network adds delays which through an excitatory-inhibitory loop would explain the promotion of oscillatory activity seen in ERNA (Steiner and Milosevic, 2023). This theory is supported by the observations that STN-DBS tends to increase firing rate in the GPe with a pattern resembling ERNA (Hashimoto et al., 2003; Reese et al., 2011), as well as pallidal-DBS producing ERNA in the pallidum of patients with PD (Johnson et al., 2023). It was also demonstrated that a computational model assembled in line with this theory was able to produce ERNA-type responses over an approximately 20 ms time period following a stimulation pulse (Schmidt et al., 2020). However, (as discussed in (Wiest et al., 2023)) extended high-frequency stimulation to the STN would likely lead to depletion of the synapses connecting the STN and GPe preventing the oscillations from continuing over an extended period of time (functional disconnection) (Bergman, 2021). This would not allow for the persistent high frequency and high amplitude ERNA remaining after multiple minutes of stimulation observed in data. To address this shortcoming of the patterned STN-GPe network firing hypothesis, it was proposed that DBS may initiate orthodromic activation of terminals to the stimulated structure (Steiner and Milosevic, 2023). This activation could also lead to delayed excitation and inhibition, therefore promoting the oscillatory behaviour of ERNA over a longer period of time.

A recent modelling study was able to replicate some of the key ERNA characteristics over a time period of approximately 200 ms using a phase oscillator network (Realmuto et al., 2023). The Kuramoto network included delays between distinct sub-populations of oscillators, which would likely be a fundamental reason as to why the network was able to reproduce these evoked oscillatory responses. However, each of these sub-populations were chosen to represent separate basal ganglia structures (globus pallidus internus, ventral intermediate nucleus of the thalamus, and the STN). Hence, this model encounters the same functional disconnection contention as proposed by Bergman et al. (Bergman, 2021). The network could instead be chosen to represent multiple sub-populations of neurons within a single basal ganglia structure (e.g. the STN for STN-DBS), however, this would likely affect the time constants of inter-sub-population delays due to proximity and connectivity. Additionally, it has been demonstrated that there exists a diverse range of responses to stimulation within neural structures as DBS leads pass through both the STN (Wiest et al., 2022) and pallidum (Cleary et al., 2013). Hence, it is important to explore how closely a single population can reproduce characteristics of ERNA to avoid these contentions, as we investigated in this study. However, when considering STN-DBS, the existence of functional connectivity in the STN has been disputed (Steiner et al., 2019). We explore the compatibility of intrinsic connectivity of the single population model with perturbation data in Supplementary Material Section E.1.

Other computational models have looked at responses evoked by DBS (Basu et al., 2018; Detorakis et al., 2015). These studies closely reproduce characteristics of ERNA fast dynamics for approximately 0.5 to 2 s following the immediate initialisation of stimulation. They achieve this through including multiple structures with delays. We instead focus on reproducing long-term ERNA dynamics over multiple minutes, which had yet to be explored. Additionally, we further expand on the work of previous models by focusing on a model of a single structure, the STN. This avoids the reliance on delays from the network to produce the resonant activity, which would likely experience synaptic failure over this longer time scale (Bergman, 2021). This has not been previously considered in models due to a focus on short-term responses. In fact, we build synaptic vesicle depletion into the model, which is a fundamental reason the model can reproduce the long-term ERNA dynamics. Additionally, by focusing on a single structure we reduce the number of model parameters which will help to prevent overfitting the model to data. We go beyond these previous studies by modelling ERNA over longer periods of stimulation, and in response to a wider variety of stimulation paradigms to validate our approach.

### 4.2 Proposed computational model

Across the many ERNA characteristics discussed in this study, we provide a preliminary validation for the modified Kuramoto model across multiple states and inputs. The model demonstrates that it has the required versatility to replicate the frequency shift between off and on-stimulation states as well as the slow dynamics of the spectrogram. The model provides a close reproduction of the frequency decay, despite being a simplification of the neuronal system that it is representing. Furthermore, we attempt to biophysically ground and validate the model.

#### 4.2.1 Validation with variable neuromodulation

The model can replicate the frequency and amplitude behaviours for variable inputs and states. In some cases, biophysically-motivated parameter changes are made by manual parameter tuning to match the initial and final frequency values in each of these conditions. However, these parameter changes also provide amplitude responses similar to the data without using these responses as features for fitting. The parameter tuning is restricted to one parameter and only by a small factor, which does not change the fundamental model dynamics. No tuning was required for the variable stimulation frequency example which provided the closest replication of the data for both the frequency and amplitude responses.

#### 4.2.2 Importance of multiple vesicle pools

The key modification of the computational model that enables the replication of long-term ERNA characteristics is the introduction of synaptic vesicle dynamics with multiple vesicle pools. The results (Fig. 2, Fig. 4, Fig. 5) appear to demonstrate frequency decays with at least two rates, in addition to the very fast RRP dynamics. We interpret these different rates of frequency decay as the initial depletion of the RP within the first 5 s, followed by depletion of the RtP in the remainder of the stimulation window (see Fig. 1B). The RRP plays a crucial role in determining the frequency of the network immediately before and after stimulation onset as well as in the post stimulation recovery.

#### 4.2.3 Considerations for other neural structures and across patients

Recent publications have shown that ERNA is not solely a property of the STN (Johnson et al., 2023), however the frequency response can be quite different between structures. In structures where ERNA is not observed (for instance in the visual cortex (Sinclair et al., 2019)) vesicle depletion is still expected to be present. We speculate that the other key properties of the model are the likely factors preventing these structures from producing ERNA (i.e. not having the high natural frequency of the neurons and a coupling function similar to Fig. 2E). Similarly, if we take the model to represent small patches of the STN rather than the entire STN, variations in model parameters pertaining to the neurons' frequency distribution and the coupling function would lead to a spatial distribution of ERNA throughout the STN.

### 4.3 Model predictions for further validation

This study has compared model predictions to previously published ERNA data, and in this section we highlight predictions corresponding to conditions for which experimental ERNA data is not available at the time of writing.

#### 4.3.1 Only one neuronal population's dynamics are required to produce ERNA

The model used in this study only requires one neuronal structure with no reciprocal feedback between different structures. Long-term recordings from the STN disconnected from the pallidum in animal models would elucidate the role of these inter-structure connections and their necessity to ERNA. Furthermore, recordings of synaptic activity between the STN and GPe after persistent STN-DBS would indicate whether functional disconnection or vesicle depletion is present.

#### 4.3.2 Vesicle depletion may be necessary for long-term dynamics of ERNA

Vesicle depletion was one of the fundamental additions to the model. Vesicle pool sizes can be estimated experimentally (Rizzoli and Betz, 2005), and the evolution of the number of vesicles at synaptic terminals during continuous deep brain stimulation (cDBS) could be compared to the vesicle depletion time-course predicted by our model. Alternatively, pharmacologically modulating vesicle re-uptake will modify the time constants of vesicle depletion. This in turn is expected to modulate the time constants of ERNA frequency decay, which could provide support for the role of vesicle depletion in the slow ERNA dynamics.

#### 4.3.3 Key features required for coupling function

While methods to experimentally estimate PRCs are well established (Nabi and Moehlis, 2011), estimating coupling functions between neurons is an area of active investigation (Gardella et al., 2016). As an alternative to estimating the coupling function, a micro electrode study of multiple STN neurons could be used to track firing times. Observing neuron firing times grouped into several clusters off stimulation would provide support for a coupling function with several stable zero-crossing points.

### 4.4 Study limitations

#### 4.4.1 Fast dynamics of vesicle depletion

The model in its current form is unable to replicate the characteristics of ERNA that are associated with the fast dynamics of vesicle depletion (see Supplementary Material sections C.1 and C.2). The model parameters were fitted to the characteristics of ERNA slow dynamics, which had previously eluded all modelling attempts. It is therefore not surprising that the model's fast dynamics response does not line up as closely with the data as the long-term characteristics. In the data, we see growing inter-pulse evoked potential amplitude over the first 10 pulses before the amplitude steadies. Following termination of stimulation, the high amplitude evoked potentials behave similarly to a damped oscillation returning to its pre-stimulation levels over around 20 ms (Sinclair et al., 2018). We can capture some, but not all of the characteristics of the fast dynamics with our model. In particular, the inter-pulse evoked potentials do not decay between pulses and appear far more sinusoidal and noise-free than the data. Furthermore, oscillations following stimulation offset are not damped (see Supplementary Material Section C.1 for more details).

The model produces higher frequency evoked potentials with low-frequency stimulation, unlike the data which only showed high-amplitude, low-frequency responses to low-frequency stimulation (Ozturk et al., 2021; Amaya et al., 2023). We demonstrate that if vesicle depletion did not occur with low-frequency stimulation, we would observe results similar to the data (see Supplementary Material Section C.2). We speculate that our current model does not replenish synaptic vesicles fast enough between pulses, which prevents us from capturing both the response to low-frequency stimulation, and the fast dynamics of ERNA as described above. In (Wiest et al., 2023), there was little change in ERNA properties over time on adaptive deep brain stimulation (aDBS), unlike cDBS where ERNA properties change before reaching steady state. It was hypothesised that this could have represented different synaptic statuses due to the ability of synaptic pools to recover while on aDBS. Therefore, there may be more complicated synaptic dynamics in the early stages following stimulation onset. Modelling the biophysical details of the RRP dynamics, including the recycling of other vesicle pools into the RRP, may provide valuable versatility to the dynamics of the model and allow the model to reproduce these features.

#### 4.4.2 Blind validation of the model

This study provides no fully blind validation of the modified Kuramoto model to the ERNA data. Despite reproducing a wide breadth of ERNA characteristics, the authors were not blinded to any of the data (even the characteristics used for validation, such as amplitude response in the variable neuromodulation sections). This is why this study is presented as a theoretical neuroscience investigation with predictions noted for future validation in other studies. However, we still manage to replicate a wide range of previously observed ERNA characteristics, a number of which could not be replicated by more complex previously published models.

#### 4.4.3 Glutamatergic/GABAergic properties of the coupling function

The fitted second-order coupling function is strongly negatively skewed (Fig. 2E). This makes the biophysical interpretation of the coupling function more complex if considering a single, isolated population describing the STN, given that it is glutamatergic (Bolam et al., 2009; Worbe et al., 2010). However, even in the off-stimulation state the oscillators synchronise into clusters, indicating a glutamatergic tendency of the network. We interpret the negative shift of the coupling function as a phase indiscriminate GABAergic input from the GPe to the STN. Additionally, as the coupling function fitted to STN data corresponds to glutamatergic units receiving GABAergic inputs, our model does not currently account for ERNA in the globus pallidus. However, while short-term ERNA has been reported in the globus pallidus in response to pallidal stimulation (Johnson et al., 2023), data on the long-term dynamics of ERNA in the globus pallidus is not publicly available at the time of writing to the best of our knowledge. We were thus unable to infer the coupling function corresponding to the GABAergic globus pallidus.

#### 4.4.4 Other biophysical simplifications

We only simulate 50 coupled phase oscillators representing a larger neuronal network responsible for ERNA. While the number of phase oscillators is lowered for computational efficiency, the network's behaviour approximates that of a larger neuronal population. We simulated the model with 200 oscillators to ensure that the model predictions are stable with respect to the size of the network. There was no fundamental changes to the model predictions with this significantly larger network. Moreover, we consider a spatially homogeneous network, and do not model the spatial variation of ERNA throughout the STN in this study. Additionally, we do not consider the effect of different types of neurotransmitters. Instead, we only model the impact of total vesicle availability on coupling strength. The interplay of multiple types of neurotransmitters and their dynamics between stimulation pulses may have to be considered to provide a more accurate representation of the oscillatory behaviour. Furthermore, the STN receives dynamic inputs from the GPe, cortex and other areas of the brain. Although we demonstrate here that only modelling one population's dynamics is sufficient to replicate the slow dynamics of ERNA, including inputs from other neuronal structures may be key to reproducing the fast dynamics of ERNA.

## 5 Conclusion

This work demonstrates that it is possible to replicate the long-term characteristics of ERNA by only considering the dynamics of a single neuronal structure. While the proposed computational model remains to be validated using follow-up data, this work has the potential to provide insights into the underlying mechanisms of ERNA. Biophysiological properties of the model key to reproduce ERNA are revealed to be vesicle depletion, properties of the coupling function, and high natural frequency of individual oscillators. These properties may inform future investigations into the underlying mechanisms of ERNA.

## CRediT authorship contribution statement

**James J. Sermon:** Writing – review & editing, Writing – original draft, Visualization, Validation, Software, Methodology, Investigation, Formal analysis, Conceptualization. **Christoph Wiest:** Writing – review & editing, Methodology. **Huiling Tan:** Writing – review & editing, Supervision, Funding acquisition. **Timothy Denison:** Writing – review & editing, Supervision, Funding acquisition. **Benoit Duchet:** Writing – review & editing, Visualization, Validation, Supervision, Software, Methodology, Investigation, Funding acquisition, Formal analysis, Conceptualization.

## Declaration of competing interest

TD is a founder, director, and shareholder of Amber Therapeutics, Ltd., which also has a controlling interest in Bioinduction Ltd. and Finetech Medical Ltd., the respective designers and manufacturers of the Picostim DyNeuMo system. TD is also an advisor for Synchron and Cortec Neuro.

## Data Availability

No data was used for the research described in the article.

## Appendix A Supplementary data

Supplementary material The supplementary material provides further details pertaining to the fitted model parameters, the model dynamics, the shortcoming of the model, fitting robustness, comparison to STN recordings, and alternative parameters with lower natural frequency. Supplementary material
