# Supplementary material for: Evoked resonant neural activity long-term dynamics can be reproduced by a computational model with vesicle depletion
Source: Neurobiol Dis. 2024 Sep;199:106565. doi: 10.1016/j.nbd.2024.106565 (PMC11300885; doi:10.1016/j.nbd.2024.106565)
Supplement: Supplementary material — The supplementary material provides further details pertaining to the fitted model parameters, the model dynamics, the shortcoming of the model, fitting robustness, comparison to STN recordings, and alternative parameters with lower natural frequency. [file mmc1.pdf]

Supplementary material

A Fitted Model Parameter Set

The results of the fitting process (outlined in the main text Section 2.5) are presented in Table A. All model parameters presented in the top row of Table A were optimisable parameters for the fitting procedure. Some of the parameters were additionally subject to manual parameter tuning in order to provide model dynamics that resembled the data more closely. At this stage, it was more efficient for an expert human to fine-tune the parameters than to run further optimisations. However, this represented only small adjustments by comparison to their fitted value. The bottom row of parameters in Table A are parameters of the three-vesicle pool model and were largely determined from data as detailed below, with a small amount of manual parameter tuning within the bounds set by the data.

Parameter	k_μ	I	ζ	f_0	f_1	f_2	f_3	f_4	Ω	σ
Value	11976	72053	5.920	-0.780	0.198	0.302	0.851	0.998	249	26.317
Parameter	M_{RRP}	M_{RP}	M_{RtP}	τ_{RRP}	τ_{RP}	τ_{RtP}	p_{RRP}	p_{RP}	p_{RtP}	
Value	1	0.5	0.3	1	10	50	0.3	0.02	0.00035	

Table A: Fitted model parameter set. The base model parameter set for all simulations. The bottom row are parameters of the three-vesicle pool model.

The time constant of each pool τ_{pool} is estimated using the approximate time required to replenish the vesicle pool through recycling in the absence of unphysiological stimulation. The RRP recycles quickly, on the order of seconds [Rizzoli and Betz, 2005], we therefore chose τ_{RRP} to be 1 second. The RP also recycles quickly on the order of seconds [Rizzoli and Betz, 2005] and given the size difference between the RRP and the RP, τ_{RRP} is taken to be 10 seconds. The RtP takes longer to replenish recycling on the order of minutes [Rizzoli and Betz, 2005], τ_{RRP} is therefore chosen to be 50 seconds.

The probability of vesicle release by a stimulation pulse p_{pool} is estimated from the time taken to deplete each vesicle. The RRP is expected to deplete within 10 pulses of unphysiological stimulation [Kaeser and Regehr, 2017], the RP over seconds and the RtP over minutes [Rizzoli and Betz, 2005]. The value of p_{pool} is taken to be inversely proportional to the number of pulses required to deplete each pool. The proportionality constant is set in combination with τ_{pool} according to the stable occupancy of each pool on 130Hz stimulation. Therefore, p_{RRP} is given a value of 0.3

(corresponding to 9 pulses on 130Hz stimulation), p_{RP} a value of 0.02 (corresponding to one second on 130Hz stimulation or 130 pulses) and p_{RtP} a value of 0.0035 (one minute or 7800 pulses) to align with these observations.

The estimated contribution of each vesicle pool on coupling strength, M_{pool}, is modelled as proportional to the product of the relative size of each vesicle pool (N_{pool}) and the inverse of the vesicle pools distance from the synaptic terminal (d_{pool}),

$$M_{\text{pool}} = \eta \frac{N_{\text{pool}}}{d_{\text{pool}}}, \tag{S.1}$$

where η is a normalising factor, the same across vesicle pools.

The RRP is the smallest vesicle pool, usually approximated to be between $1-2\%$ of the overall number of vesicles [Rizzoli and Betz, 2005]. Additionally, RRP vesicles are usually considered to be either docked or very close to the synaptic terminal. Given the approximate size of vesicle pools around 20nm [Südhof, 2012, Dittman and Ryan, 2019] in diameter, we estimate the average distance of RRP vesicles to the synaptic terminal to be on the scale of 10nm. The important consideration for N_{pool} is the relative size between pools. We therefore take the total number of vesicles as 100 and taking the percentage of vesicles in the RRP as 1%, we obtain $N_{\text{RRP}}/d_{\text{RRP}} = 0.1\text{nm}^{-1}$. We choose the normalising factor $v = 10\text{nm}$ (across all pools), which gives $M_{\text{RRP}} = 1$. The RP contains slightly more vesicles at around $10-20\%$ [Rizzoli and Betz, 2005], but is located further from the synaptic terminal. Furthermore, the RP must release fewer vesicles to the synapse when full compared to the RRP. Electron microscopy and neurotransmitter release simulations suggests that the majority of vesicles located proximal to the synapse are located within 500nm of the terminal [Rizzoli and Betz, 2005, Branco and Staras, 2009, Qu et al., 2009, Dittman and Ryan, 2019], therefore we estimate the average distance of the RP vesicles to the synaptic terminal as 200nm. Taking the percentage of vesicles in the RP as 10%, we obtain $N_{\text{RP}}/d_{\text{RP}} = 0.05\text{nm}^{-1}$ and $M_{\text{RP}} = 0.5$. Lastly, the RtP represents the majority of vesicles, $80-90\%$ [Rizzoli and Betz, 2005] and given the percentages taken for the other vesicle pools, we will assume the RtP represents 89%. Estimates for the furthest distance vesicle pools take from the synaptic terminal are between 1µm [Dittman and Ryan, 2019] and 5µm [Rizzoli and Betz, 2005, Staras et al., 2010], we therefore take an approximate distance of 3µm. We obtain $M_{\text{RtP}} = 0.3$.

B Explaining Model Dynamics

B.1 Amplitude Response

One of the benefits of using computational models is that we can investigate the dynamics that may be responsible for the ERNA characteristics in the simulations. Here we investigate what causes the amplitude variation seen over the longer time period. By recording the phase difference between one random oscillator against all the others, we can observe clustering behaviour over both the off- and on-stimulation periods (Fig S.1). Fig S.1Bi shows that in the steady off-stimulation state we see two

clusters approximately anti-phase (due to the coupling function, see Fig 2E), which results in low amplitude activity. In on-stimulation histograms of Figs S.1Bii, iii and iv, it can be seen that there is a shift away from an even distribution between each cluster, with a preference to synchronise to one cluster. The development of this distribution of phases between clusters over the stimulation window accounts for the amplitude variation. Firstly, the histograms of Figs S.1Biii and iv do not differ greatly as can be seen from the amplitude dynamics with little variation after approximately 50 seconds of stimulation. In the early part of the stimulation window there is greater coupling around a single cluster at an approximate ratio of 25:1 oscillators in each group compared to the smaller cluster. In the later part of the stimulation window, this ratio shrinks to around 10:1 as coupling strength drops further and there is less attraction to stay coupled to the dominant cluster.

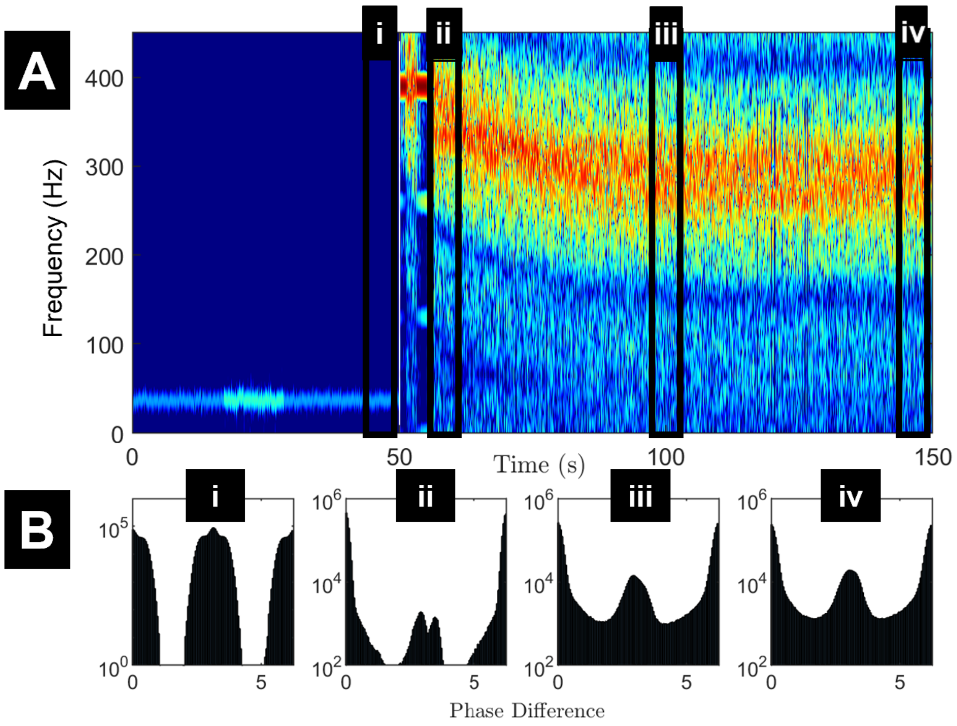

Figure S.1: Clustering of oscillators explains the amplitude dynamics over continuous stimulation. (A) Spectrogram of network activity (Same as in Fig 2A) over 50 seconds off-stimulation before turning on-stimulation for 100 seconds. The black boxes labelled i, ii, iii and iv refer to the periods clustering was observed over for the histograms in panel B. (B) Histograms of phase differences for four five second periods. (i) 44 to 49 seconds, (ii) 55 to 60 seconds (to avoid brief period of entrainment), (iii) 97.5 to 102.5 seconds, and (iv) 144 to 149 seconds. Panel Bi is plotted with vertical axis limits of 10^0 to 10^6, whereas panels Bii-iv are plotted with limits of 10^2 to 10^6, to demonstrate the lack of activity observed outside the clusters.

B.2 Frequency Response

The on-stimulation frequency response can be explained by investigating the dynamics of individual oscillators as well. After each stimulation pulse, the oscillators are pushed towards synchronisation around a single phase due to the sinusoidal PRC. This would occur for any PRC with a stable zero-crossing point. Furthermore, this occurs regardless of the length of time following stimulation onset as this is a property that is independent of vesicle depletion's effect on coupling strength. Early in the stimulation window, before vesicle depletion has reduced coupling strength too greatly, the oscillators remain mostly synchronised around a single phase for the majority of the remaining inter-pulse interval (see Fig S.1Bii). Given that coupling strength is still high and that the coupling function is positive for zero phase difference, this increases the frequency of the network at this early stage. Later in the stimulation window, as coupling strength reduces, fewer oscillators remain synchronised and for a shorter period of time (see Fig S.1Biii and iv), resulting in less phase advancement from the positive coupling function for zero phase difference. Moreover, the low synchronisation does not allow entrainment to stimulation [Sermon et al., 2024]. Hence, the primary effect on oscillator phase progression comes from the natural frequency of the individual oscillators (Eq.1). Therefore, the network trends to a frequency close to the fitted mean of the natural frequencies of all the oscillators in the network after a long period of high frequency stimulation (see $\Omega = 249$ in Table A).

C Current Model Shortcomings

C.1 Fast Dynamics

For comparison to the fast ERNA dynamics reported in [Sinclair et al., 2018], DBS is turned on at 130Hz for a period of 100ms with 50ms of observations off-stimulation either side of the DBS window. This follows an initial 10 seconds of the model settling. Initially coupling strength decayed on-stimulation following our three-vesicle pool model with the parameters obtained from the fitting process (Table A). Coupling strength begins to recover with a one-second time constant as dictated by RRP recovery. However, high frequency and high amplitude dynamics are maintained during the 100ms following stimulation termination due to coupling strength remaining low at this time scale (Fig S.2). Model dynamics return to low frequency conditions within around one second following stimulation termination (see for example Fig 3B). We also note that inter-pulse dynamics are very regular and do not resemble typical ERNA signals. Hence, our fitted model reproduces the slow dynamics of ERNA, but is unable to capture its fast dynamics. This may be due to the slow replenishment of vesicles in our simplified model on this time scale. Introducing more complex dynamics, such as vesicle mixing between pools or STN-GPe excitatory/inhibitory connections, may be necessary to reproduce fast ERNA dynamics.

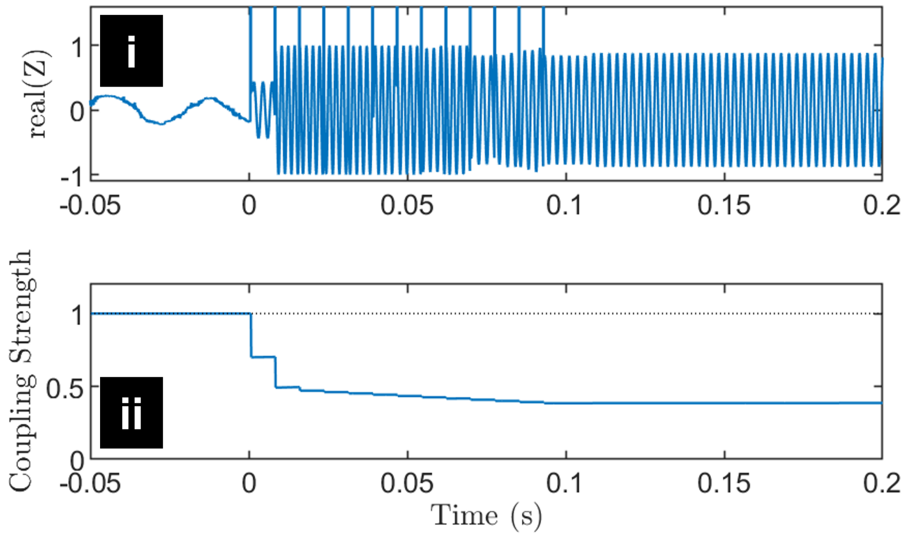

Figure S.2: Fast dynamics of ERNA for 100ms of 130Hz stimulation pulses. (i) The approximate amplitude response with stimulation artefact artificially superimposed on the signal following the simulation for demonstration purposes. (ii) The corresponding coupling strength of the network.

C.2 Subtherapeutic Stimulation

Subtherapeutic stimulation at 20Hz can elicit evoked potentials [Ozturk et al., 2021]. Similar responses were also seen at lower stimulation frequencies of 3Hz [Zapata Amaya et al., 2023]. These evoked potentials had increased amplitude compared to baseline, but no high frequency content. 20Hz stimulation may be considered on the borderline of physiological-unphysiological stimulation [Denker and Rizzoli, 2010], while 3Hz stimulation is certainly within the physiological limits. However, this is dependent on the neural structure being stimulated and 20Hz may even be considered within physiological frequencies in some structures, especially if this stimulation frequency is close to the natural frequency of the neuronal ensemble.

The application of low frequency stimulation to our model does not align with these results, however. For both 3Hz and 20Hz we still see the promotion of high frequency activity (Figs S.3A and E). Stimulation at 3Hz produces evoked responses at lower frequencies compared to higher stimulation frequencies tried. Hints from literature can help us investigate this shortcoming further. Stimulation at 10Hz has been shown to not cause vesicle depletion in some cases [Fernández-Alfonso and Ryan, 2004]. Hence, it is likely that little vesicle depletion will be observed in response to 20Hz stimulation. Furthermore, as 20Hz stimulation elicits very similar responses to 3Hz stimulation [Zapata Amaya et al., 2023], it could be expected that these two stimulation paradigms elicit similar neural responses. Hence, we investigate the evoked response in the absence of vesicle depletion for 20Hz stimulation (Fig S.3I), otherwise the model and parameters remain unchanged.

Without vesicle depletion in the model, we do not see the evolution of high frequency activity (see Figs S.3I and L). However, we still see the promotion of high amplitude activity, in line with [Ozturk et al., 2021, Zapata Amaya et al., 2023] (see

Fig S.3K). This is primarily due to the maintenance of a higher coupling strength from the lack of vesicle depletion. Therefore, it is likely that improving the fast dynamics of the model as suggested in the previous section (especially with regards to the dynamics of the RRP) would make the model behave more similarly to data in response to subtherapeutic stimulation.

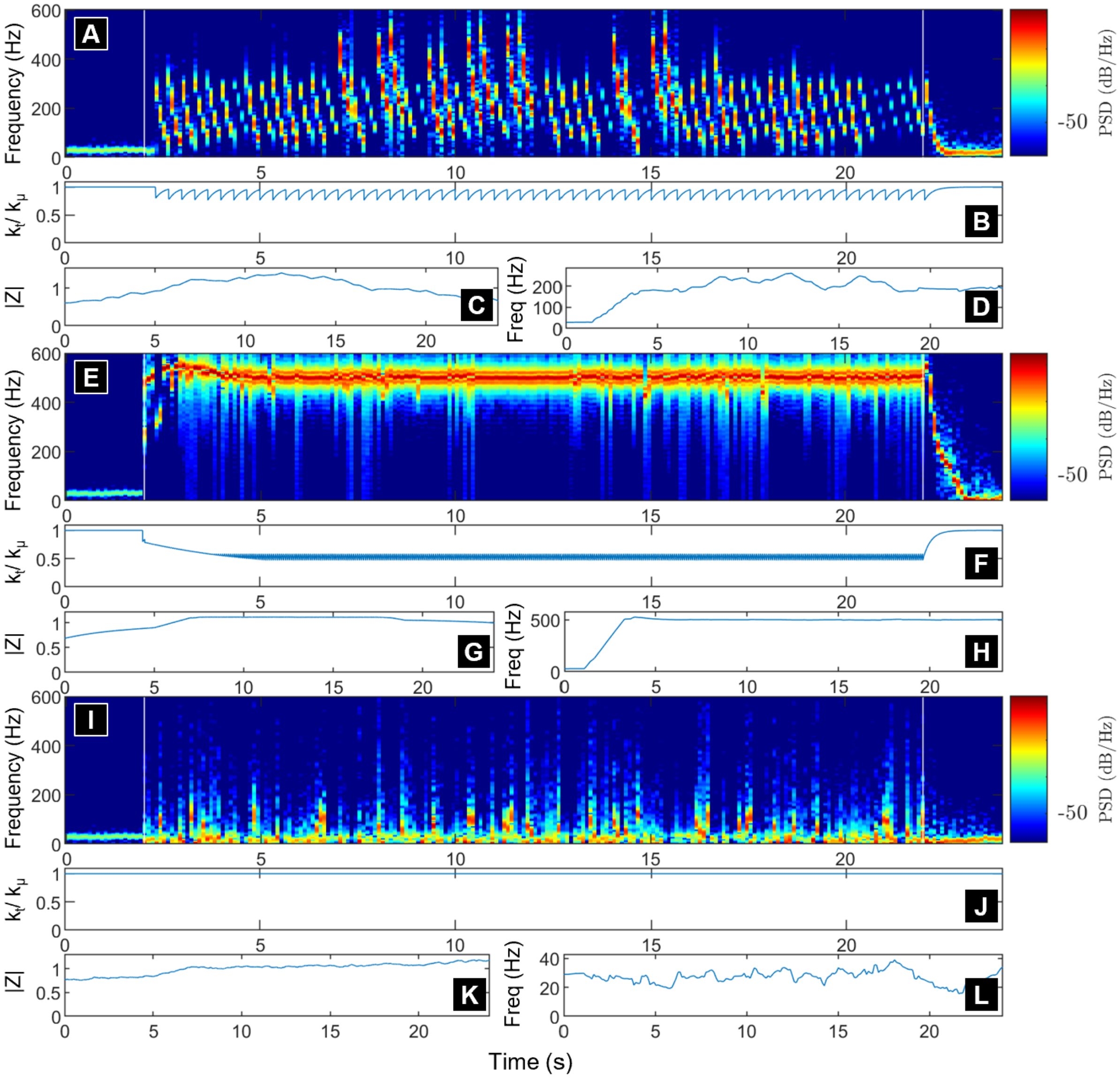

Figure S.3: Response of the model to low frequency stimulation with and without vesicle depletion as a theoretical mechanism of subtherapeutic stimulation. (A, E and I) Spectrogram with stimulation frequency at 3Hz (A) and 20Hz (E and I). Stimulation onset at two seconds into simulation indicated by white line. (B, F and J) The evolution of coupling strength for each simulation. (C, G and K) Amplitude of oscillations over whole simulation. (D, H and L) Peak frequency response for each of the 100ms windows over the whole simulation. (A-H) Vesicle depletion consistent with Eqs. 4 and 5. (I-L) No vesicle depletion.

D Fitting Robustness

Here we explore how changing the coupling function and the PRC in the fitted model affects the ERNA response to stimulation.

D.1 Sensitivity of Coupling Function

A second-order Fourier series coupling function was able to capture more complex neuronal interactions than the sine function commonly used in the Kuramoto model. As shown in the main text, fitting the Fourier series coefficients provided slow dynamics closely approximating the ERNA spectrogram. For comparison we also simulate the model with a sinusoidal coupling function and a coupling function derived from the Hodgkin-Huxley neuron model [Nabi and Moehlis, 2011], while all other parameters remain constant (see Fig S.4). These coupling functions do not lead to ERNA dynamics (Figs S.4B and C). Additionally, the dynamics of the sine coupling function result in activity at a much higher frequency off stimulation (around 200-250Hz), and at a much higher amplitude (Fig S.4B). This is due to simpler clustering behaviour compared to the fitted coupling function, which favours two clusters in the absence of stimulation. The sine coupling function does not give rise to changes in oscillation frequency with stimulation despite vesicle depletion remaining present (Figs S.4B). The Hodgkin-Huxley derived coupling function shows a response where frequency increases with time on-stimulation and vesicle depletion (Figs S.4C). While these results are based on model parameters obtained for the second-order Fourier coupling function, attempts to fit to the long-term ERNA frequency decay using each of these coupling functions were made, but no promising results were obtained.

The Fourier series fitted coupling function is a key element of the model to reproduce the ERNA frequency decay, therefore we also investigate the sensitivity of the five fitted coefficient values. For each of the Fourier series coefficients we re-simulate the computational model after modulating the coefficients by the factors 0, 0.25, 0.5, 1, 2, and 4 (Fig S.5). The columns corresponding to the first-order coefficients (f_1 and f_2) show little variability for all factors (including 0) both on- and off-stimulation. Hence, the first order coefficients are not only not very sensitive, but may not be necessary for the model. The other three coefficients all seem to produce more notable changes to the spectrograms when modulated. The changes result in either failing to reproduce off-stimulation dynamics, on-stimulation dynamics, or both such that the resulting spectrogram no longer matches all characteristics of the slow ERNA dynamics.

D.2 Sensitivity of Phase Response Curve

We also compare different types of PRCs (Fig S.6). We scale the Hodgkin-Huxley PRC to a maximum amplitude of one to provide a comparable scale to the sinusoidal PRC. We consider a constant PRC of magnitude $1/\sqrt{2}$. These PRCs are chosen so that we can identify the role of the PRC in the computational model. As the constant PRC does not replicate the on-stimulation dynamics, it is likely that a

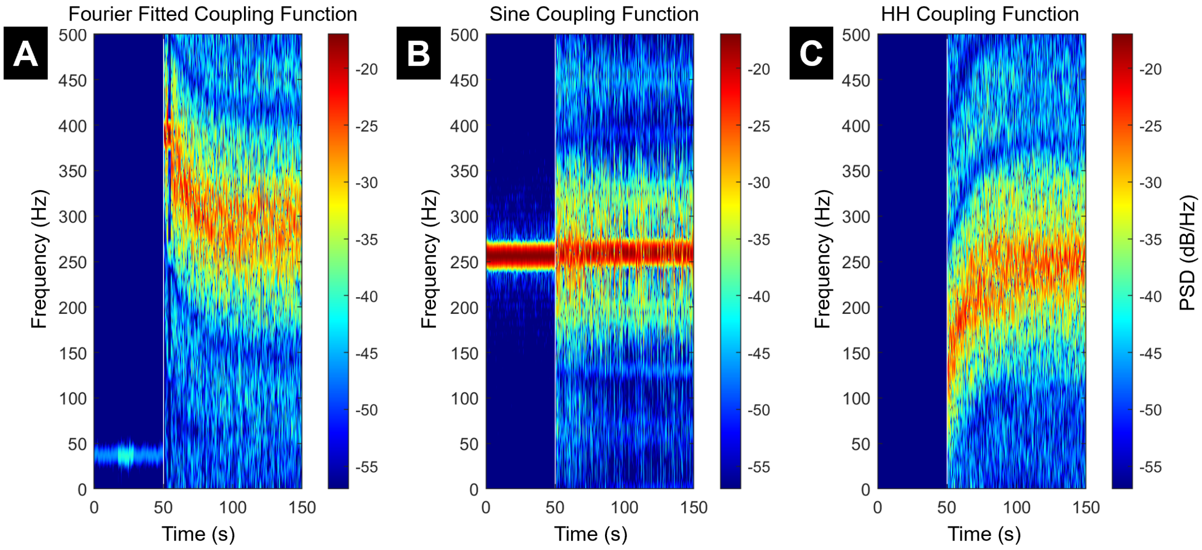

Figure S.4: Effect of the coupling function on ERNA slow dynamics. 50 seconds off-stimulation followed by 200 seconds on-stimulation. (A) The fitted second-order Fourier series coupling function used in the rest of the study, similar figure to Fig 2A. (B) Sinusoidal coupling function. (C) Hodgkin-Huxley (HH) derived coupling function. Other than coupling function changes, all other model parameters remain the same.

synchronising effect from the PRC is required to reproduce ERNA. This agrees with the results from Section B.1. The Hodgkin-Huxley PRC (Fig S.6B) is chosen as a more biophysical representation, and we do see the peak of the frequency decay start at 390Hz (the third harmonic of stimulation frequency) before dropping down. There is intermittent locking at the 5:2 harmonic (325Hz for 130Hz stimulation frequency) before dropping further and locking to the second harmonic of stimulation (at 260Hz). However, unlike the sine PRC and the data, the locking to components of stimulation frequency is much stronger to the point where activity only seems to appear at these components. This could be due to the sharper gradient of the Hodgkin-Huxley PRC at the zero-crossing point having a stronger synchronising effect on the oscillators than the sinusoidal PRC. As the fitting was performed using the sinusoidal PRC, it is certainly possible that by fitting with the Hodgkin-Huxley PRC instead we could observe a response that more closely resembles the data.

E Further Simulations for Comparison to STN Recordings

We perform simulations of the model analogous to previously published in vitro and in vivo STN recordings to further test the model against biophysical data.

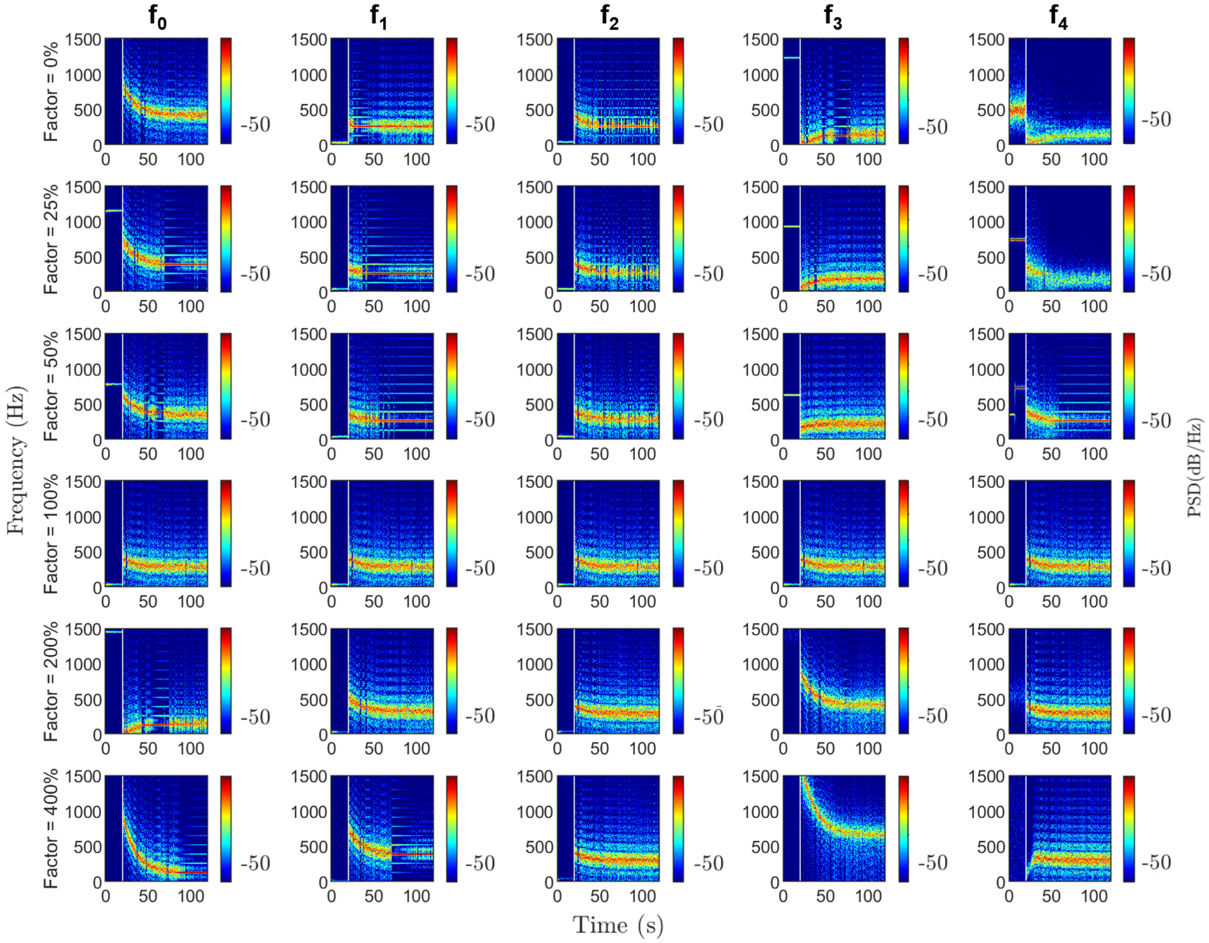

Figure S.5: Effect of modulating each Fourier coefficient of the coupling function on ERNA slow dynamics. Spectrograms showing 20 seconds off-stimulation followed by 100 seconds on-stimulation, observed over the large frequency range of [0Hz,1500Hz] due to high frequency activity in some of the simulations. Columns ordered by Fourier coefficients: f_0 correspond to the vertical shift, f_1 and f_2 are coefficients corresponding to the first harmonic components (cosine and sine, respectively), and f_3 and f_4 are coefficients corresponding to the second harmonic components (cosine and sine, respectively). Rows ordered by the factor multiplying the coefficients. Factor of 100% represents no change to the coefficient with respect to the fitted results, so this row has the same parameters for each of the coefficient simulations.

E.1 Individual Oscillator Perturbation

While coupling between STN neurons (and how it varies over stimulation) is crucial in our model's ability to reproduce ERNA dynamics, results presented in [Steiner et al., 2019] portray the STN as a mostly disconnected network of neurons. Steiner et al. showed that stimulating individual STN neurons to the point of firing does not cause neighbouring STN neurons to fire. We therefore simulate perturbations to single oscillators in the network to determine whether these results can be replicated in the model while still having connectivity between STN neurons. We individually perturb seven of the oscillators from our network (all-to-all connected) with four pulses

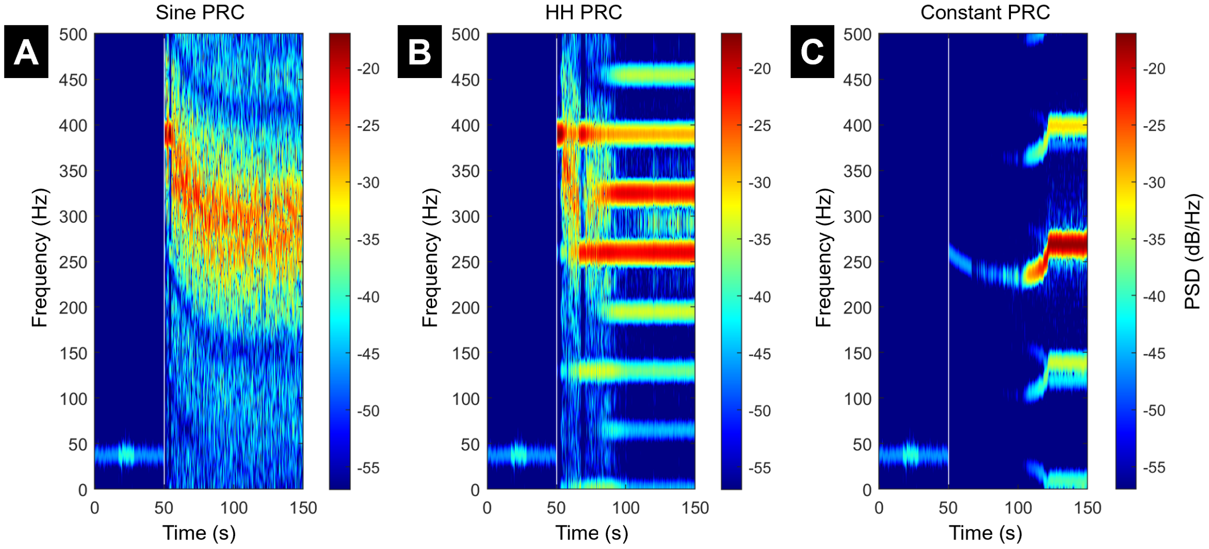

Figure S.6: Effect of the PRC on ERNA slow dynamics. 50 seconds off-stimulation followed by 200 seconds on-stimulation. (A) sinusoidal PRC, similar simulation to Fig 2A. (B) Hodgkin-Huxley (HH) PRC. (C) Constant PRC. Other than PRC changes all parameters remain the same as the main body of this study.

at 130Hz and the same stimulation amplitude as the model was fitted to (3mA). In [Steiner et al., 2019], four pulses at 20Hz were used, in this study we proceed with 130Hz as the model did not capture the long term 20Hz response (see Section C.2). This is a conservative choice, since 130Hz stimulation elicits faster phase progression of the oscillators in the model. We record the rate of phase progression in all seven oscillators with each perturbation and compare this against the phase progression of the oscillators in the absence of stimulation to any part of the network. There appears to be no marked change in phase progression rate of the oscillators across the network in response to a single oscillator being perturbed (Fig S.7). All of the oscillators not being directly stimulated respond similarly showing a small increase in phase progression, but not enough to experience any additional firing ($< 2\pi$, corresponding to one cycle). This is seen across the network regardless of the oscillator being perturbed or observed.

E.2 Replication of In Vitro Slice Experiments

The behaviour of mostly disconnected oscillators in the model matches data from in vitro slice recordings of the rat STN (Fig S.8). Rat STN neurons in slices were shown to fire around 20Hz off-stimulation [Hallworth et al., 2003, Wilson et al., 2004]. In the presence of constant current electrical stimulation, firing rate increases rapidly to over 200Hz at low stimulation amplitude (<0.5nA) [Hallworth et al., 2003, Wilson et al., 2004], up to over 500Hz in some cases [Kitai and Kita, 1987]. This was consistent with high-frequency pulsatile stimulation (>120Hz, 200μA) where firing rate of individual STN neurons was seen to increase to over 200Hz [Schor et al., 2022].

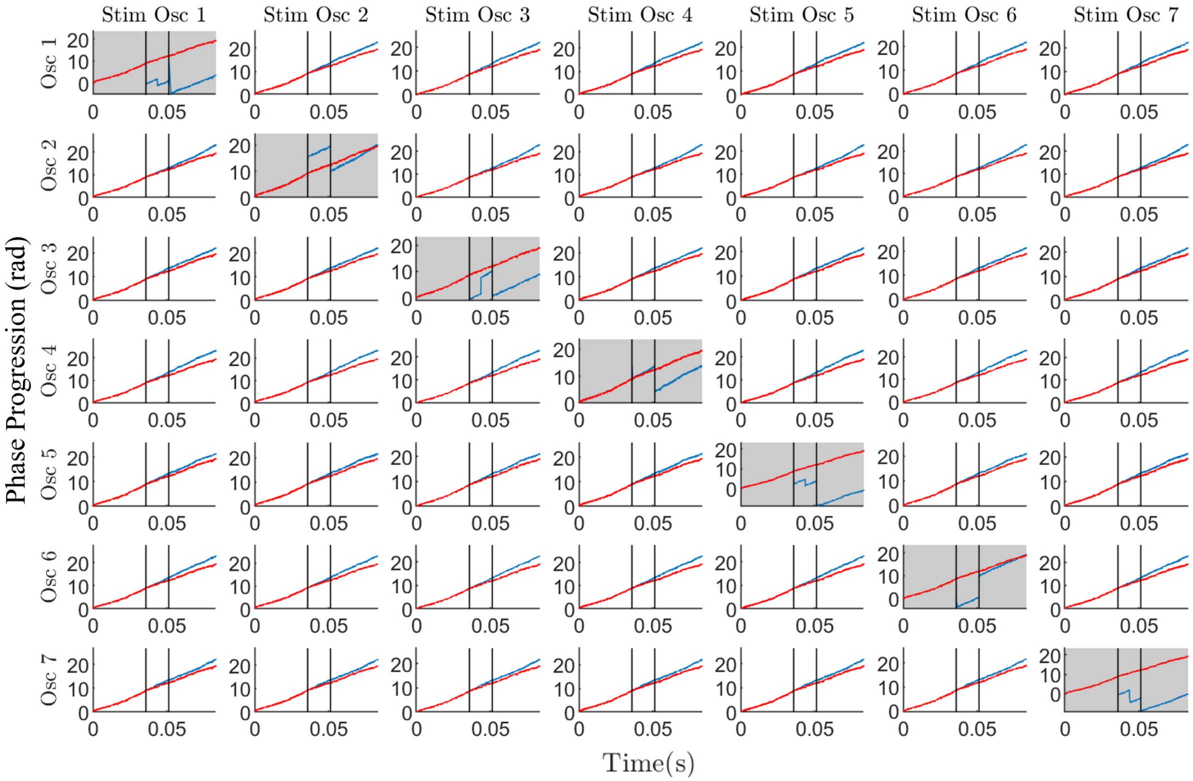

Figure S.7: The phase progression of individual oscillators in response to the perturbation of a single oscillator in the network for comparison against data [Steiner et al., 2019]. Observing phase progression of oscillator indicated by row title during stimulation of oscillator indicated by column title. The grey panels represent a case where we are recording the phase progression from the same oscillator as it is being stimulated. White panels indicate a recording from an oscillator not receiving microelectrode equivalent stimulation. The red lines on each plot represent the phase progression of each oscillator (number indicated by row heading) in the absence of stimulation to any oscillator in the network. The blue lines represent the phase progression of the oscillator in response to stimulation of a single oscillator. Stimulation is applied at 130Hz for four pulses at the fitted stimulation amplitude between the two vertical black lines as was done in the data [Steiner et al., 2019].

We aim to reproduce firing rates of STN neurons explanted from rats exceeding 250Hz with constant current stimulation [Kitai and Kita, 1987, Hallworth et al., 2003, Wilson et al., 2004]. These in vitro slices represent neurons mostly disconnected from the rest of the STN. To replicate this condition in the model we reduce the network down to the fewest number of oscillators for which coupling can exist, two oscillators. In order to capture the absence of GABAergic input to the slice after removal from the brain, we increase the shift of the coupling function between the two oscillators. We adjust the shift of the coupling function so that both oscillators progress with a frequency of approximately 20Hz (f_0 from -0.780 to -0.110 in Eq (6)) (see Fig S.8) as seen in the off-stimulation data [Kitai and Kita, 1987, Hallworth et al., 2003, Johnson et al., 2023]. Constant direct current stimulation is then applied. The stimulation amplitude is adjusted from the value the full model was fitted to by a

factor $130 \times \Delta t = 0.013$ to deliver the same stimulation intensity per oscillator in this simulation as in the 130Hz pulsatile stimulation case in the full model. We approximate the instantaneous frequency of the ith oscillator at time t as

$$\nu_{i,t} = \frac{\theta_{i,t+1} - \theta_{i,t}}{2\pi \Delta t}, \tag{S.2}$$

which is then smoothed (moving average, 100ms window) to reduce the effect of noise.

Following the application of constant current stimulation, the two oscillators progress at a frequency greater than 500Hz immediately following stimulation onset (Fig S.8B). The stimulation amplitude used was likely higher than the slice experiments used, as the model was fitted to pulsatile stimulation data of around 3mA. Despite the likely disparity in the amplitude of stimulation, the firing rate on-stimulation matches the observations of neuronal firing rate increasing to over 500Hz with stimulation [Kitai and Kita, 1987]. When vesicle depletion is included in this network, the approximate frequency of the oscillator firing rate stabilises around 300Hz.

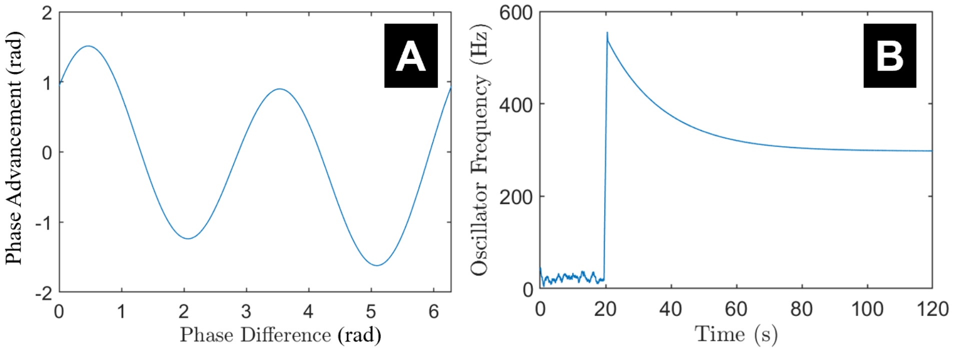

Figure S.8: The approximate frequency of individual oscillators in a two-oscillator network with the adjusted coupling function and constant current stimulation for comparison of mostly disconnected in vitro slice experiments. (A) Adjusted coupling function after changing noughth order coefficient. (B) Individual oscillator frequency in the two-oscillator network over constant stimulation current injection window of 100s following onset after 20 seconds off-stimulation.

F Alternative Parameter Set with Lower Natural Frequency

We explore the modified Kuramoto model further by constraining the natural frequencies of individual oscillators to lower frequencies. Results from STN slice experiments could indicate that individual STN oscillators have natural frequencies around 20Hz. Previously, we assumed that 20Hz was the frequency of the overall neural ensemble and did not reflect individual neuron properties, therefore not con-

straining the ω_i's in the model. Here, we constrain the mean natural frequency of oscillators (Ω) to 20Hz. Given the model set up, it is possible to maintain similar off-stimulation dynamics by countering the decrease in natural frequency by a corresponding increase in the noughth order shift of the coupling function f_0 (see values in Table B).

Parameter	Ω	σ	f_0	p_{RtP}	τ_{RtP}
Values for Fig S.9	20	2.11	-0.68	0.00035	50
Values for Fig S.10	20	2.11	-0.68	0.00007	10

Table B: Alternative parameter sets for low natural frequency simulations. All other parameters remain unchanged from Table A.

Following the adjustments to natural frequency and f_0, we simulate the model while keeping all other parameters the same as the fitted model (Fig S.9). We see similar amplitude dynamics decreasing over the stimulation window (Fig S.9C) as with the fitted model. However, as coupling strength continues to decrease over the stimulation window, we see the frequency decay reach much lower values towards the end of the stimulation window (Fig S.9B). It continues to decrease to a value lower than that seen from the ERNA data (<100Hz). Fig S.9A shows two short periods of entrainment at stimulation frequency (130Hz) around 40 seconds into the simulation. This is similar to Fig 2A, but we observe entrainment at a lower harmonic due to the lower oscillating frequency of the network in this adjusted simulation.

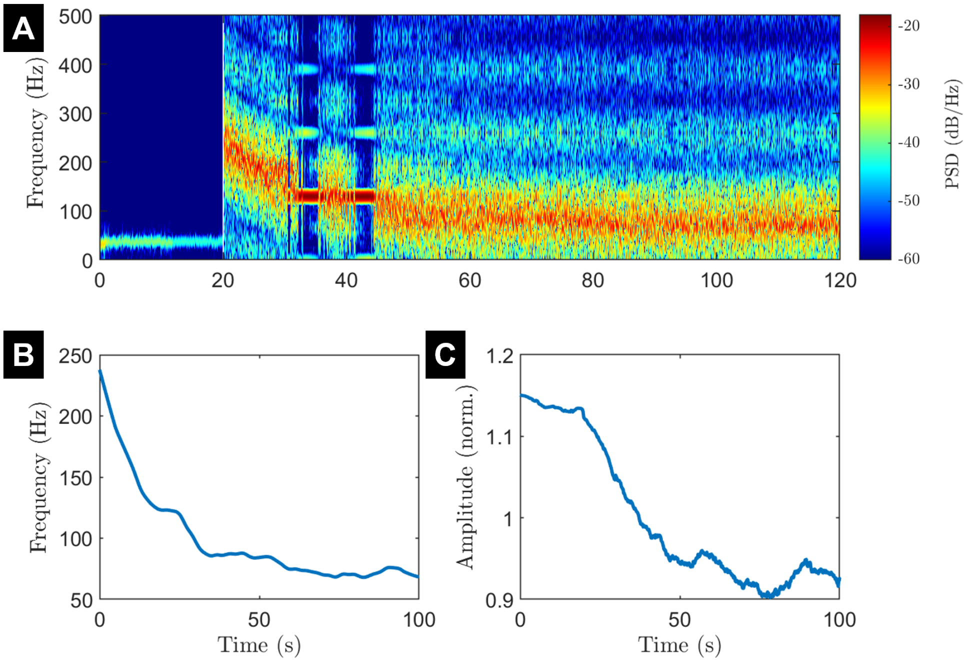

Figure S.9: Slow dynamics for lowered natural frequency model with all other parameters remaining the same. (A) The spectrogram of 20 seconds off-stimulation followed by 100 seconds on-stimulation. (B) The on-stimulation frequency decay. (C) the on-stimulation amplitude response.

Given how vesicle depletion changes the coupling strength during stimulation, it is not possible to maintain the same dynamics on-stimulation with this same method. Hence, to maintain a similar frequency decay, the long term dynamics of the RtP (as dictated by τ_{RtP} and p_{RtP}) are modulated (see second row of Table B) such that the frequency at the end of the 100-second stimulation window approximately matches that of the fitted modified Kuramoto model from the main part of this study (Fig 2)A. While it is possible to maintain higher frequency dynamics (Fig S.10B), the amplitude decay does not demonstrate any substantial variation, remaining somewhat constant (Fig S.10C). Hence, to see amplitude variation it may be required that RtP occupancy and therefore coupling strength decreases low enough such that oscillators can move between clusters. By reducing coupling strength closer to zero we allow the network to oscillate at a similar frequency to the natural frequency of the individual oscillators. While it might be possible to achieve this with an alternative resolution of the coupling function, we have explored this hypothesis in detail. We have repeated the fitting process with constrained natural frequencies to 20Hz and have not observed encouraging results.

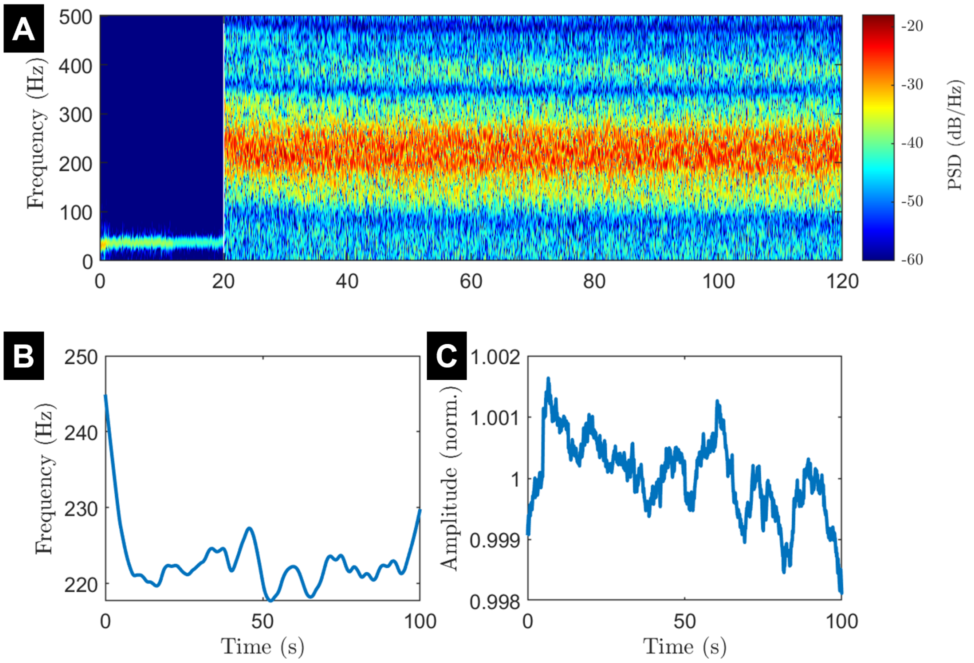

Figure S.10: Slow dynamics for lowered natural frequency model with coupling strength changed to approximately fit to frequency decay. (A) The spectrogram of 20 seconds off-stimulation followed by 100 seconds on-stimulation. (B) The on-stimulation frequency decay. (C) the on-stimulation amplitude response.

References

[Branco and Staras, 2009] Branco, T. and Staras, K. (2009). The probability of neurotransmitter release: Variability and feedback control at single synapses. *Nature Reviews Neuroscience*, 10.

[Denker and Rizzoli, 2010] Denker, A. and Rizzoli, S. O. (2010). Synaptic vesicle pools: An update. *Frontiers in Synaptic Neuroscience*.

[Dittman and Ryan, 2019] Dittman, J. S. and Ryan, T. A. (2019). The control of release probability at nerve terminals. *Nature Reviews Neuroscience*, 20.

[Fernández-Alfonso and Ryan, 2004] Fernández-Alfonso, T. and Ryan, T. A. (2004). The kinetics of synaptic vesicle pool depletion at cns synaptic terminals. *Neuron*, 41.

[Hallworth et al., 2003] Hallworth, N. E., Wilson, C. J., and Bevan, M. D. (2003). Apamin-sensitive small conductance calcium-activated potassium channels, through their selective coupling to voltage-gated calcium channels, are critical determinants of the precision, pace, and pattern of action potential generation in rat subthalamic nucleus neurons in vitro. *Journal of Neuroscience*, 23.

[Johnson et al., 2023] Johnson, K. A., Cagle, J. N., Lopes, J. L., Wong, J. K., Okun, M. S., Gunduz, A., Shukla, A. W., Hilliard, J. D., Foote, K. D., and Hemptinne, C. D. (2023). Globus pallidus internus deep brain stimulation evokes resonant neural activity in parkinson's disease. *Brain Communications*, 5.

[Kaeser and Regehr, 2017] Kaeser, P. S. and Regehr, W. G. (2017). The readily releasable pool of synaptic vesicles. *Current Opinion in Neurobiology*, 43.

[Kitai and Kita, 1987] Kitai, S. T. and Kita, H. (1987). Anatomy and physiology of the subthalamic nucleus: A driving force of the basal ganglia. In Carpenter, M. B. and Jayaraman, A., editors, *The Basal Ganglia II*, pages 357–373, Boston, MA. Springer US.

[Nabi and Moehlis, 2011] Nabi, A. and Moehlis, J. (2011). Single input optimal control for globally coupled neuron networks. *Journal of Neural Engineering*, 8.

[Ozturk et al., 2021] Ozturk, M., Viswanathan, A., Sheth, S. A., and Ince, N. F. (2021). Electroceutically induced subthalamic high-frequency oscillations and evoked compound activity may explain the mechanism of therapeutic stimulation in parkinson's disease. *Communications Biology*, 4.

[Qu et al., 2009] Qu, L., Akbergenova, Y., Hu, Y., and Schikorski, T. (2009). Synapse-to-synapse variation in mean synaptic vesicle size and its relationship with synaptic morphology and function. *Journal of Comparative Neurology*, 514.

[Rizzoli and Betz, 2005] Rizzoli, S. O. and Betz, W. J. (2005). Synaptic vesicle pools. *Nature Reviews Neuroscience*, 6.

[Schor et al., 2022] Schor, J. S., Montalvo, I. G., Spratt, P. W., Brakaj, R. J., Stansil, J. A., Twedell, E. L., Bender, K. J., and Nelson, A. B. (2022). Therapeutic deep brain stimulation disrupts movement-related subthalamic nucleus activity in parkinsonian mice. *eLife*, 11.

[Sermon et al., 2024] Sermon, J. J., Starr, P. A., Denison, T., and Duchet, B. (2024). Pre-existing oscillatory activity as a condition for sub-harmonic entrainment of finely tuned gamma in Parkinson's disease. *Brain Stimulation: Basic, Translational, and Clinical Research in Neuromodulation*, 17(2):488–490.

[Sinclair et al., 2018] Sinclair, N. C., McDermott, H. J., Bulluss, K. J., Fallon, J. B., Perera, T., Xu, S. S., Brown, P., and Thevathasan, W. (2018). Subthalamic nucleus deep brain stimulation evokes resonant neural activity. *Annals of Neurology*, 83:1027–1031.

[Staras et al., 2010] Staras, K., Branco, T., Burden, J. J., Pozo, K., Darcy, K., Marra, V., Ratnayaka, A., and Goda, Y. (2010). A vesicle superpool spans multiple presynaptic terminals in hippocampal neurons. *Neuron*, 66.

[Steiner et al., 2019] Steiner, L. A., Tomás, F. J. B., Planert, H., Alle, H., Vida, I., and Geiger, J. R. (2019). Connectivity and dynamics underlying synaptic control of the subthalamic nucleus. *Journal of Neuroscience*, 39.

[Südhof, 2012] Südhof, T. C. (2012). The presynaptic active zone. *Neuron*, 75.

[Wilson et al., 2004] Wilson, C. J., Weyrick, A., Terman, D., Hallworth, N. E., and Bevan, M. D. (2004). A model of reverse spike frequency adaptation and repetitive firing of subthalamic nucleus neurons. *Journal of Neurophysiology*, 91.

[Zapata Amaya et al., 2023] Zapata Amaya, V., Aman, J. E., Johnson, L. A., Wang, J., Patriat, R., Hill, M. E., MacKinnon, C. D., Cooper, S. E., Darrow, D., McGovern, R., Harel, N., Molnar, G. F., Park, M. C., Vitek, J. L., and Escobar Sanabria, D. (2023). Low-frequency deep brain stimulation reveals resonant beta-band evoked oscillations in the pallidum of parkinson's disease patient. *Frontiers in Human Neuroscience*, 17.
